# Identification of Prognostic Genes Related to Cell Senescence and Lipid Metabolism in Glioblastoma Based on Transcriptome and Single-Cell RNA-Seq Data

**DOI:** 10.3390/ijms26051875

**Published:** 2025-02-21

**Authors:** Qiong Li, Hongde Liu

**Affiliations:** State Key Laboratory of Digital Medical Engineering, School of Biological Science and Medical Engineering, Southeast University, Nanjing 211189, China; ql814495@gmail.com

**Keywords:** glioblastoma, cell senescence, lipid metabolism, single-cell RNA sequencing, prognostic genes

## Abstract

Glioblastoma (GBM) is the most aggressive primary brain cancer, with poor prognosis due to its aggressive behavior and high heterogeneity. This study aimed to identify cellular senescence (CS) and lipid metabolism (LM)-related prognostic genes to improve GBM prognosis and treatment. Transcriptome and scRNA-seq data, CS-associated genes (CSAGs), and LM-related genes (LMRGs) were acquired from public databases. Prognostic genes were identified by intersecting CSAGs, LMRGs, and differentially expressed genes (DEGs), followed by WGCNA and univariate Cox regression. A risk model and nomogram were constructed. Analyses covered clinicopathological features, immune microenvironment, somatic mutations, and drug sensitivity. GBM scRNA-seq data identified key cells and prognostic gene expression. SOCS1 and PHB2 were identified as prognostic markers, contributing to the construction of a robust risk model with excellent predictive ability. High-risk group (HRG) patients had poorer survival, higher immune and stromal scores, and distinct somatic mutation profiles. Drug sensitivity analysis revealed significant differences in IC50 values. In microglia differentiation, SOCS1 and PHB2 showed dynamic expression patterns. These findings provide new strategies for GBM prognosis and treatment.

## 1. Introduction

Glioblastoma (GBM), also known as glioblastoma multiforme, is the most common brain cancer in the central nervous system (CNS) and is a grade IV glioma [1]. Its morbidity and mortality rates are high. Statistics show that there are approximately 5.36 new cases per 100,000 people, and the five-year mortality rate exceeds 90% [2]. Currently, the clinical treatment of GBM mainly relies on surgical intervention, supplemented by postoperative radiotherapy and adjuvant chemotherapy [3]. However, even with the standard Stupp treatment regimen, which combines maximal safe resection, radiotherapy, and temozolomide chemotherapy, the median survival of GBM patients remains less than two years, highlighting the urgent need for more effective therapeutic strategies [4]. Although various treatment strategies, such as radiotherapy, chemotherapy, and immunotherapy, are still being developed and applied, the prognosis of GBM patients is still poor, with a median survival of only 15 months [5,6]. Factors such as the blood–brain barrier (BBB), immune escape, tumor heterogeneity, and tumor resistance may be the main reasons for the poor treatment effect of GBM. The BBB is a major obstacle to effective GBM treatment, limiting the penetration of therapeutic agents into the brain parenchyma. Composed of tight junctions between endothelial cells, pericytes, and astrocytes, the BBB restricts the diffusion of macromolecules, including most chemotherapeutic agents and biologics, significantly reducing the efficacy of systemic therapies [7]. However, BBB-associated drug resistance remains a significant hurdle in GBM therapy. Furthermore, immune evasion mechanisms enable GBM to circumvent host immune surveillance [8]. GBM cells employ multiple immunosuppressive strategies, including the overexpression of immune checkpoint molecules such as programmed death-ligand 1 (PD-L1), the secretion of immunosuppressive cytokines such as transforming growth factor-beta (TGF-β) and interleukin-10 (IL-10), and the recruitment of regulatory T cells (Tregs) and myeloid-derived suppressor cells (MDSCs) [9]. Furthermore, GBM exhibits extensive heterogeneity and lacks universal target genes, complicating treatment and leaving its mechanisms of invasion and immune escape poorly understood [10]. In addition, GBM also shows strong resistance to radiotherapy and chemotherapy, especially by suppressing T cell activity through immunosuppressive molecules, thus inducing resistance to immunotherapy [11]. Given the high morbidity and mortality of GBM, further research into its molecular mechanisms and potential prognostic markers is essential for early diagnosis and improved treatment strategies.

Cellular senescence (CS) is a permanent cell cycle arrest mediated by the p53/p21CIP1 and/or p16INK4A/Rb signaling pathways, characterized by senescence-associated secretory phenotype (SASP), anti-apoptotic program, and lysosomal content. The increase significantly affects cell metabolism [12]. In the development of cancer, CS can be triggered by multiple factors, including DNA damage, oncogene activation, therapeutic drugs, or an increase in reactive oxygen species (ROS). CS exerts growth inhibitory effects in damaged cells and is often associated with cancer and the aging process [13,14]. Inducing cancer cell senescence can not only limit tumor growth but also activate immune cells through factors released by senescent cells, thereby triggering the regression of cancer [15]. However, there is a complex interrelationship between CS and the development of GBM and its response to treatment. Continuous stimulation of cancer cells can lead to T cell senescence and exhaustion, thereby triggering dysregulated immune responses [16,17]. Therefore, exploring the complex interactions between GBM and CS-related genes (CSAGs) is particularly important for the treatment of GBM.

Metabolic reprogramming is a hallmark of GBM and plays a crucial role in tumor growth, survival, and therapeutic resistance [18]. While glucose and amino acid metabolism have been extensively studied in GBM, recent evidence suggests that lipid metabolism also contributes significantly to tumor progression [19]. Lipids serve as essential components of cell membranes, energy sources, and signaling molecules, enabling tumor cells to adapt to metabolic stress and evade therapeutic interventions [20]. Abnormal expressions of genes related to lipid metabolism may promote the growth and invasion of GBM [21,22]. Therefore, understanding the role of lipid metabolism in GBM is critical for identifying new therapeutic targets, as an in-depth study of the mechanisms of lipid metabolism and lipid metabolism-related genes (LMRGs) in GBM can provide valuable insights into the biological characteristics of the tumor and facilitate the discovery of novel treatment strategies.

There is a close connection between cell senescence and lipid metabolism. Cell senescence not only changes the metabolic state of cells but also may regulate the tumor microenvironment (TME) by affecting the expression of genes related to lipid metabolism. Studies have shown that changes in lipid metabolism in senescent cells can affect the cytokines they secrete, thereby affecting the behavior of surrounding cells [23]. In GBM, the interaction between senescence-related genes and LMRGs may play an important role in tumor progression and immune escape [24]. Therefore, exploring the relationship between cell senescence and CSAGs and lipid metabolism and LMRGs in GBM will provide a new perspective for understanding this complex disease.

Single-cell sequencing technology provides a powerful tool for the in-depth study of the mechanism of action of CSAGs and LMRGs in GBM. This technology can reveal the gene expression characteristics of different cell types, identify key genes, and clarify their specific functions in tumor progression and treatment response by analyzing the transcriptome of single cells in the tumor microenvironment. For instance, a pioneering single-cell RNA-seq study on primary GBM profiled 430 tumor cells from five patients and demonstrated that GBM cells exhibit extensive transcriptional diversity, particularly in oncogenic signaling, proliferation, immune response, and hypoxia-related pathways [25]. Recent studies utilizing scRNA-seq and flow cytometry have further characterized the immune landscape of GBM, demonstrating dynamic shifts in immune cell populations throughout tumor progression. Early-stage tumors were enriched in pro-inflammatory microglia, whereas late-stage tumors exhibited a predominance of anti-inflammatory macrophages and myeloid-derived suppressor cells (MDSCs), correlating with BBB disruption and EGFR+ tumor expansion [26]. These findings highlight the importance of single-cell sequencing in uncovering GBM heterogeneity, resistance mechanisms, and potential personalized treatment strategies [27].

Although GBM has attracted extensive attention as a highly invasive malignant tumor, the expression patterns, prognostic effects, and molecular functions of CSAGs and LMRGs in GBM are relatively scarce, and the related molecular mechanisms have not been fully elucidated. Therefore, this study aimed to evaluate candidate genes related to CSAGs and LMRGs in GBM through bioinformatics analysis, identified potential prognostic genes combined with univariate Cox and Lasso analysis, and developed a prognostic model to evaluate the potential role of prognostic genes in GBM. The study predicted functional enrichment differences, immune status, TME, and somatic mutations of GBM patients stratified by risk scores and provided new treatment options for GBM patients. In summary, this study utilized bioinformatics analysis, clinical data mining, and statistical model construction and was committed to filling the gaps in CSAGs and LMRGs in GBM research, providing new perspectives and strategies for disease mechanisms, prognostic evaluation, and the development of treatment options.

## 2. Results

### 2.1. Identification of 4468 WGCNA-Related Genes

**Figure 1:**
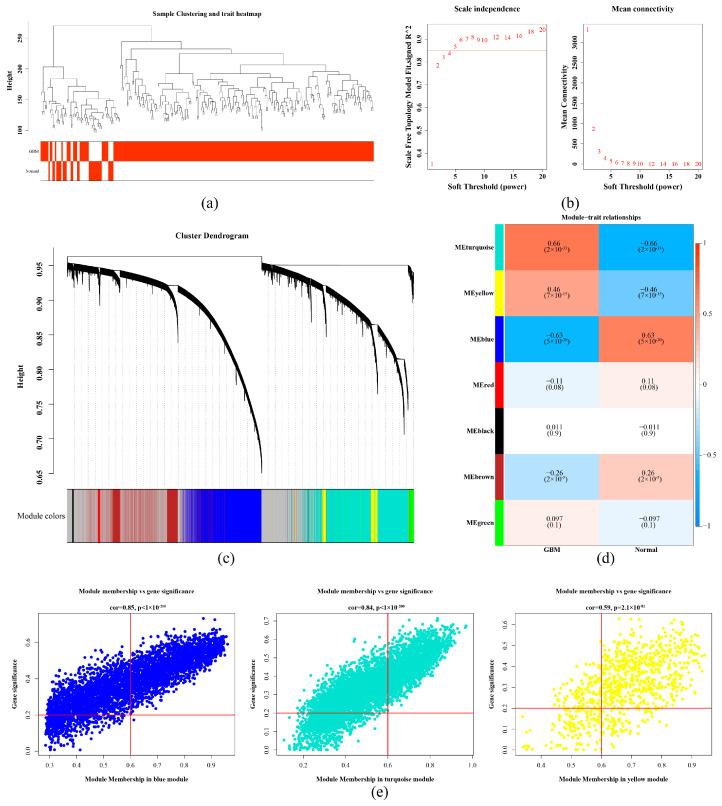
Identification of WGCNA-related genes and key modules in glioblastoma. (**a**) Sample-level clustering. Each branch in the clustering tree represents a sample, and the vertical coordinate represents the sample expression Euclidean distance. (**b**) Soft threshold screening. (**c**) Co-expression module identification. (**d**) Heatmap of correlations between key modules and non-tumor control samples, GBM samples. (**e**) The scatter plot between Gene Significance (GS) and Module Membership (MM). Meanwhile, |MM| > 0.6 and |GS| > 0.2 were used as thresholds to further screen the genes in the significant modules, in which 1816 genes were obtained from the turquoise color module, 554 genes from the yellow module, and 2098 genes from the blue module. Combined, 4468 genes were obtained.

The results of WGCNA demonstrated that there were no outlier samples (Figure 1a). The optimal soft threshold was 5 (R^2^ = 0.85), and the average connectivity of the co-expression network was close to 0 (Figure 1b). After excluding genes that could not be classified and belonged to the gray module, a total of seven co-expression modules were identified (Figure 1c). Among them, turquoise modules, yellow module, and blue module were modules that were substantially associated with GBM, with association coefficients being 0.66, 0.46, and −0.63, respectively (*p* < 0.05) (Figure 1d). After screening with thresholds of |MM| > 0.6 and |GS| > 0.2, 1816, 554, and 2098 genes were obtained, respectively (obtained Figure 1e). After combination, 4468 WGCNA-related genes were acquired (Supplementary Table S1).

### 2.2. Enrichment Functions and PPI Analysis for 15 Candidate Genes

**Figure 2:**
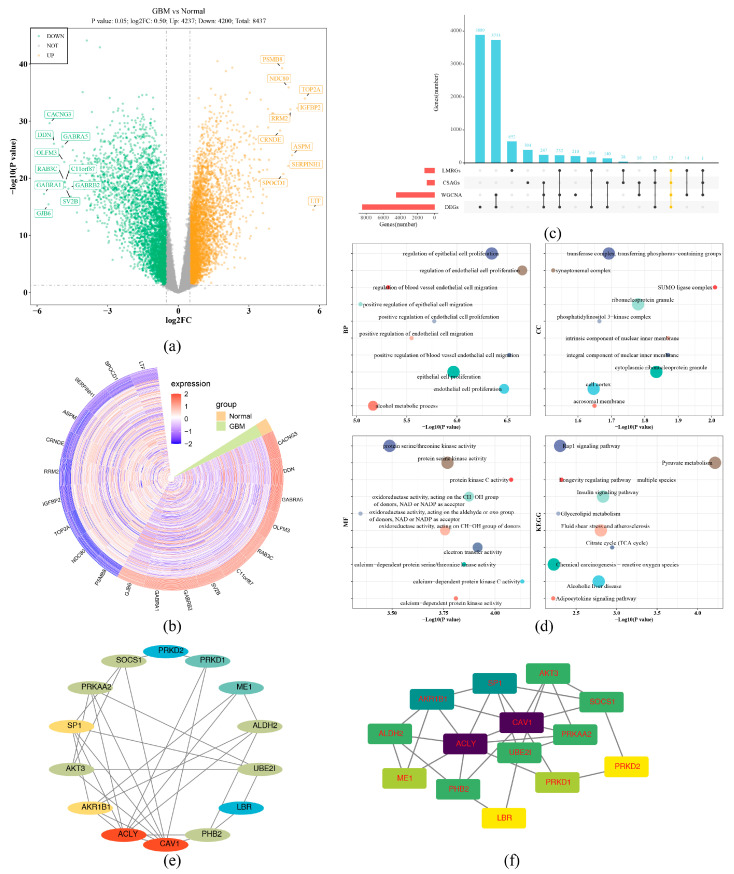
Functional enrichment and PPI network analysis of 15 candidate genes. (**a**) Differential gene volcano plots. The labeled genes in the figure are the top 10 upregulated genes and the top 10 downregulated genes with the largest log2 (Fold Change). (**b**) Heatmap of ring expression density of differential genes. Grouping bars at the end of the ring heatmap, light yellow indicates normal samples, and light green indicates GBM samples; in the heatmap, the redder the color, the higher the expression. (**c**) Upset plot of candidate selections. Colors and markers are used to highlight overlapping relationships between methods. (**d**) GO and KEGG enrichment results (top10). (**e**,**f**) Network diagram of STRING analysis of candidate genes. The PPI network showed that after removing one isolated gene, there were 14 nodes and 30 edges in the network diagram. Among these genes, AKR1B1, SP1, ACLY, and CAV1 had relatively strong interactions with other genes.

Meanwhile, 8437 DEGs were gained, among which 4237 genes were up and 4200 genes were down in GBM (Figure 2a,b, Supplementary Table S2). Finally, 15 candidate genes were obtained from 4468 WGCNA-related genes, 8437 DEGs, 866 CSAGs, and 1133 LMRGs (Figure 2c, Supplementary Table S3). A total of 571 GO terms (*p* < 0.05) were enriched by candidate genes. Among them, there were 506 BPs, such as regulation of endothelial cell proliferation, positive regulation of blood vessel endothelial cell migration, and regulation of epithelial cell proliferation; 14 CCs, like the integral component of the nuclear inner membrane, the cytoplasmic ribonucleoprotein granule, and the ribonucleoprotein granule; and 51 MFs, for example, calcium-dependent protein kinase C activity, acting on the CH-OH group of donors, with NAD or NADP as an acceptor (Figure 2d, Supplementary Table S4). Moreover, a total of 41 KEGG pathways (*p* < 0.05) were enriched by candidate genes, including pyruvate metabolism, citrate cycle (TCA cycle), and longevity regulating pathway-multiple species, etc. (Figure 2e, Supplementary Table S5). Furthermore, the PPI network showed that after removing one isolated gene, there were 14 nodes and 30 edges in the network diagram. Among these genes, AKR1B1, SP1, ACLY, and CAV1 had relatively strong interactions with other genes (Figure 2f).

### 2.3. SOCS1 and PHB2 as Prognostic Genes and Construction of Risk Model

**Figure 3:**
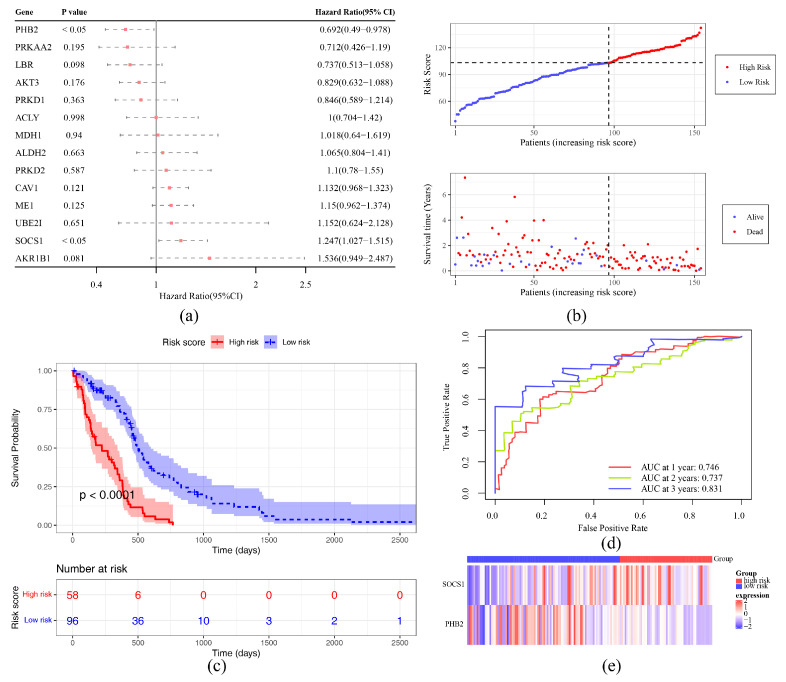
Identification of SOCS1 and PHB2 as prognostic genes and development of a risk prediction model. (**a**) Forest plot of one-factor Cox regression analysis. HR is abbreviated as risk ratio; when HR > 1, the cue factor is a facilitator of the occurrence of a positive event; when HR < 1, the cue factor is a deterrent to the occurrence of a positive event; and when HR = 1, the cue factor has no effect on the occurrence of a positive event. (**b**) Distribution of risk scores and survival status of primary tumor samples in TCGA-GBM sets. (**c**) KM curve analysis. The horizontal axis is the total survival time (days), and the vertical axis is the probability of survival; the high-risk group is shown in red, and the low-risk group is shown in blue. (**d**) ROC curve of training set. (**e**) Prognostic gene expression analysis.

**Figure 4:**
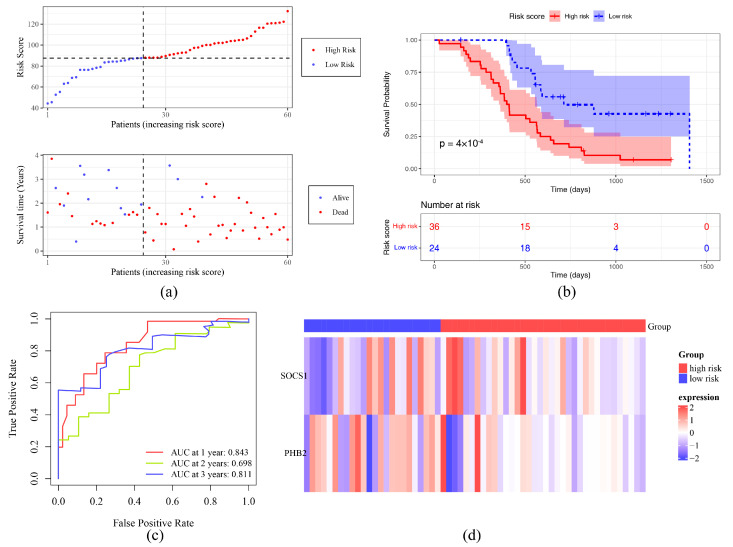
Validation of risk prediction model. (**a**) Distribution of risk scores and survival status of primary tumor samples in the validation set. (**b**) KM curve of high- and low-risk groups. (**c**) ROC curve of the validation set. (**d**) Prognostic gene expression analysis.

Two prognostic genes (SOCS1 and PHB2) were gained through univariate Cox regression analysis (*p* < 0.05) and satisfied the PH test (*p* > 0.05) (Figure 3a, Supplementary Table S6). The RSF model was founded on these prognostic genes to predict the risk score of each patient. In TCGA-GBM and validation sets, optimal cut-off values of risk score were 103.013990 and 87.568442. In addition, the survival status plot indicated that the higher the risk score, the more GBM patients who died (Figure 3b). The K–M curve showed that the survival probability of the HRG was lower than the LRG (*p* < 0.05) (Figure 3c). The ROC curve demonstrated that AUC values for 1-year, 2-year and 3 years in TCGA-GBM were 0.746, 0.737, 0.831 (Figure 3d). SOCS1 was highly expressed in the HRG, while PHB2 was expressed in the opposite direction (Figure 3e). In the validation set, the survival status plot, KM curve, and gene expression trends were consistent with the validation set, AUC > 0.7 (Figure 4). The above results indicated that the risk model had relatively good accuracy and stability in predicting prognosis of GBM patients.

### 2.4. Differences in Clinicopathological Features Between the HRG and LRG 

**Figure 5:**
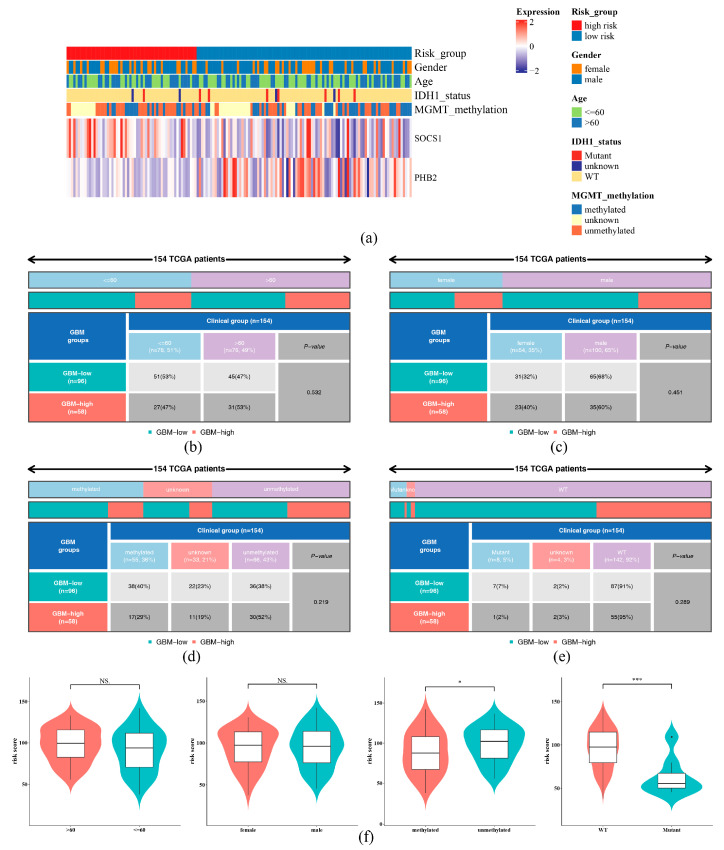
Differences in clinicopathological features between the high-risk group (HRG) and low-risk group (LRG). (**a**) Heatmap of clinical features and molecular pathology characteristics of GBM samples. The top annotations of the samples include the following clinical features: risk group (red for the high-risk group, blue for the low-risk group), sex (orange for female, blue for male), age (light green for age 60 and below, dark blue for age 60 and above), IDH1 mutation status (light yellow for wild-type, red for mutant, and dark blue for unknown), and MGMT promoter methylation status (dark blue for methylated, orange for unmethylated, and yellow for unknown). (**b**–**e**) Distribution of risk status among subgroups of different clinicopathological features (age (**b**), gender (**c**), MGMT methylation (**d**), IDH1 status (**e**)) in the HRG and LRG (*p* > 0.05). Different color codes in the figure denote low-risk (blue) and high-risk (red) samples, with the number and proportion of samples in each group and the significance *p*-value. (**f**) Correlation of Risk Scores with Clinical Characteristics. *** represented *p* < 0.001, * represented *p* < 0.05, and NS represented *p* > 0.05.

**Figure 6:**
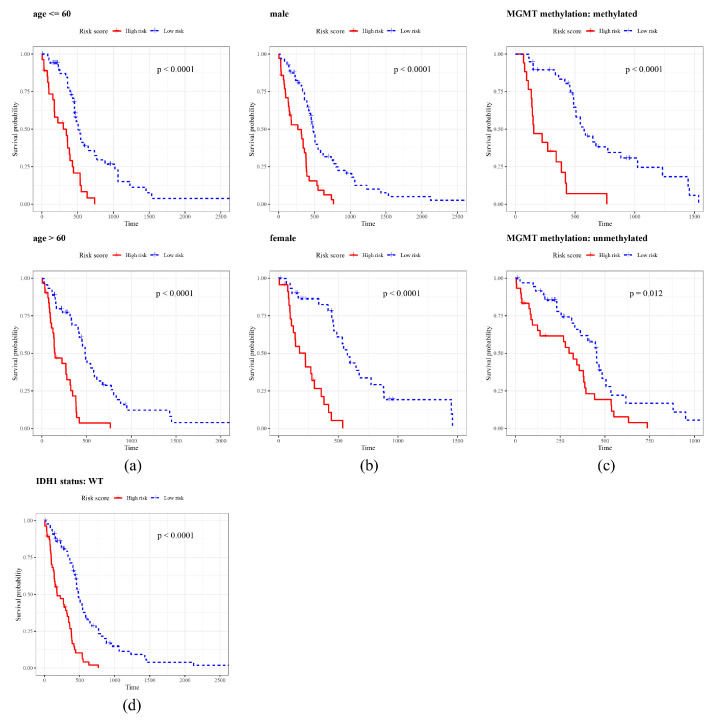
KM curve analysis. age (**a**), gender (**b**), MGMT methylation (**c**), IDH1 status (**d**). The horizontal axis is the overall survival time (days), and the vertical axis is the survival probability, with the high-risk group in red and the low-risk group in blue. The *p*-value was used to assess the statistically significant difference in survival time between the high- and low-risk groups.

The heatmap demonstrated that different clinicopathological features had distinct expression patterns in the HRG and LRG (Figure 5a, Supplementary Table S7). The expression levels of SOCS1 and PHB2 differed between the high- and low-risk groups, suggesting that these genes may have significant biological implications and potential prognostic value in GBM. There was no substantial difference in distribution of risk status among subgroups of different clinicopathological features (age, gender, IDH1 status, MGMT methylation) in the HRG and LRG (*p* > 0.05) (Figure 5b–e). However, substantial differences in risk score were observed in IDH1 status (WT higher than mutant) and MGMT (methylated higher than unmethylated) methylation subgroups (*p* < 0.05) (Figure 5f). The K–M curve showed that in each subgroup of age (≤60 years, >60 years), gender (male and female), IDH1 status (WT), and MGMT methylation (methylated and unmethylated), the survival probability of the HRG was lower than that of the LRG (*p* < 0.05) (Figure 6), indicating that patients with a high risk score had a poorer prognosis.

### 2.5. A Nomogram with Better Prognostic Value

**Figure 7:**
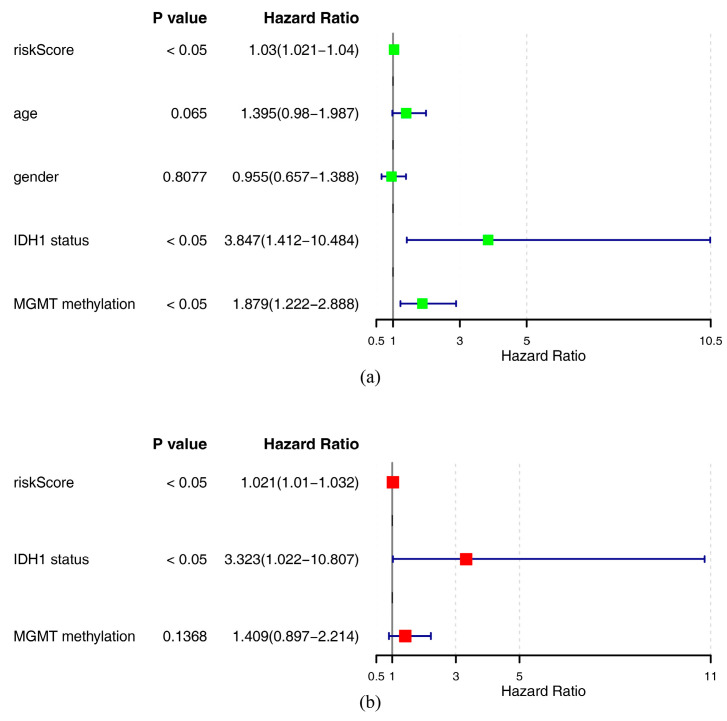
Prognostic analysis of the risk model in glioblastoma. (**a**) Results of one-way Cox analysis. (**b**) Results of multifactorial Cox analysis.

**Figure 8:**
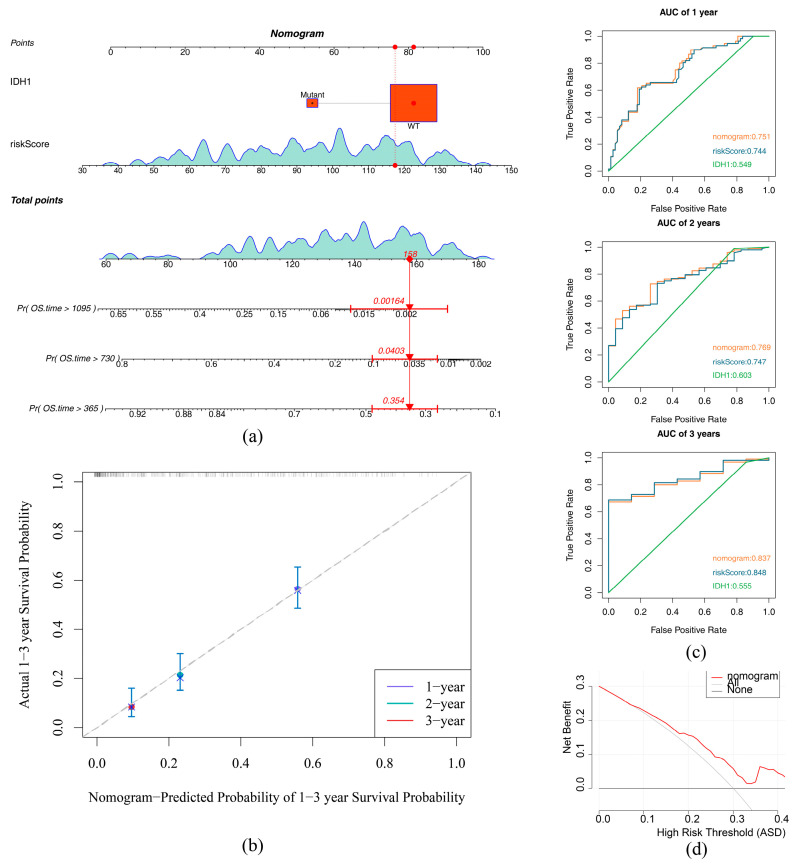
Construction and validation of the nomogram. (**a**) Nomogram. The upper half of the column line graph allows for the calculation of scores for individual factors; the lower half allows for the speculation of the probability of survival for GBM patients based on the total score obtained. (**b**) Column line graphs 1-, 2-, and 3-year calibration curves. Horizontal coordinates are predicted event rates, and vertical coordinates are observed actual event rates, both ranging from 0 to 1. (**c**) ROC curve analysis. (**d**) DCA Curve. The diagonal line (All) represents all samples with all interventions; the horizontal line (None) represents all samples with no intervention.

The IDH1 status, MGMT methylation, and risk score were obtained through univariate Cox analysis (*p* < 0.05) (Figure 7a) and obtained through a PH test (*p* > 0.05) (Supplementary Table S8). Subsequently, risk score and IDH1 mutation status were identified as independent prognostic traits through multivariate Cox analysis (Figure 7b). The nomogram formed in line with these independent prognostic factors showed that the higher the total score was, the lower the survival probability of GBM patients would be (Figure 8a). The calibration curve demonstrated that predicted survival probabilities at 1, 2, and 3 years almost coincided with reference line (Figure 8b), indicating that nomogram had relatively high prediction accuracy. The ROC curve revealed that AUC values at 1, 2, and 3 years were 0.75, 0.77, and 0.84, respectively (Figure 8c), suggesting that nomogram exhibited excellent predictive efficacy (AUC > 0.7). The DCA curve indicated that the net advantage of the nomogram model was more prominent than that of the diagonal line (all) and horizontal line (none) (Figure 8d), demonstrating that the nomogram model had good predictive performance and practical application value in clinical decision-making for GBM.

### 2.6. Functional Annotation and Immune Infiltration in the HRG and LRG 

**Figure 9:**
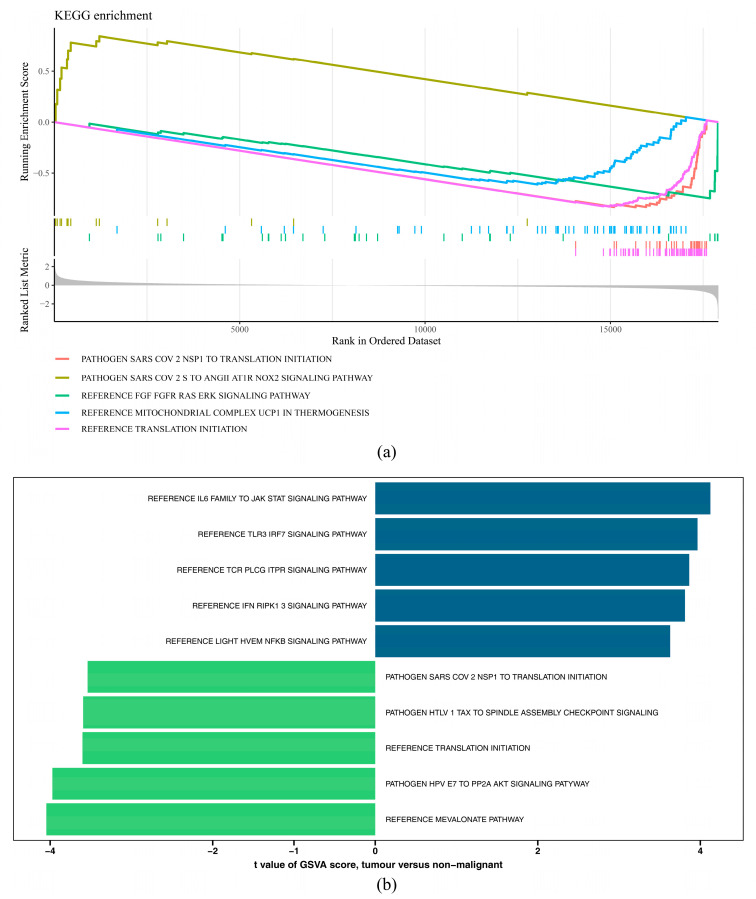
Functional prognostic genes and risk model in glioblastoma. (**a**) GSEA enrichment pathway (top5). (**b**) Results of GSVA in the high- and low-risk groups. t > 0 represents pathways that were significantly upregulated in the high-risk group, and t < 0 represents pathways that were significantly downregulated in the high-risk group.

The GSEA showed that 70 significantly enriched pathways were obtained in the HRG and LRG, such as reference translation initiation, reference FGF fgfr ras erk signaling pathway, reference mitochondrial complex ucp1 in thermogenesis, etc. (Figure 9a, Supplementary Table S9). The GSVA indicated that there were 132 significantly different pathways in the HRG and LRG. Among them, 72 pathways were markedly overexpressed and 60 pathways were substantially suppressed in the HRG. The up pathways included the reference IL6 family to JAK STAT signaling pathway, reference TLR3 IRF7 signaling pathway, reference IFN RIPK1 3 signaling pathway, etc. The down pathways consisted of the reference mevalonate pathway, pathogen HPV E7 to PP2A AKT signaling pathway, reference translation initiation, etc. (Figure 9b, Supplementary Table S9). The above results demonstrated that various signaling pathways and translation regulations played a vital function in GBM.

**Figure 10:**
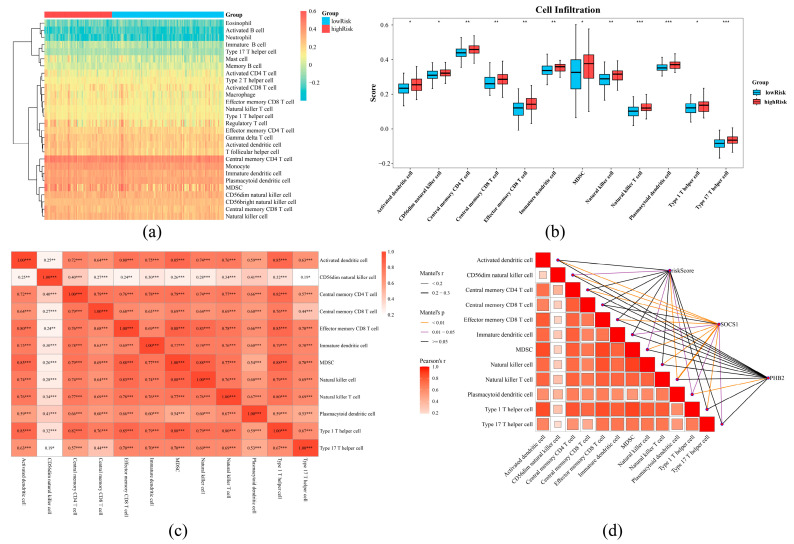
Immune infiltration analysis for high- and low-risk groups. (**a**) Immune cell abundance in samples from the high-risk and low-risk groups. The heat map colors reflect the level of immune infiltration, with higher levels of immune infiltration tending to be more red and lower levels tending to be bluer. (**b**) Differential infiltration of immune cells between the high- and low-risk groups, yellow represents the low-risk group, and red represents the high-risk group. (**c**) Differential immune infiltrating cell correlation heat map, red represents positive correlation, blue represents negative correlation, a darker color means higher correlation; * reflects the significance level, and the numbers in the boxes are correlation values. *** represented *p* < 0.001, ** represented *p* < 0.01, * represented *p* < 0.05. (**d**) Heatmap of prognostic genes, risk scores, and differential immune cell correlations; the lower left corner shows the relationship between immune cells with differential infiltration, and the magnitude of the correlation is reflected in the size and color of the squares in the matrix. The upper right line color and thickness reflect the range of *p*-values and the range of correlations, respectively.

Among the 28 types of immune cells, central memory CD4 T cells had the highest immune score (Figure 10a). And, 12 types of differential immune cells were gained, and all of them showed higher infiltration levels in the HRG, such as central memory CD4 T cells, effector memory CD8 T cells, immature dendritic cells, and natural killer cells (*p* < 0.05) (Figure 10b). Additionally, there was a positive relationship among all differential immune cells. Among them, the most substantial positive relationship was found between Type 1 T helper cells and myeloid-derived suppressor cells (MDSC) (cor = 0.89, *p* < 0.05) (Figure 10c). The most significant positive relationship was observed between SOCS1 and natural killer T cells (cor = 0.55, *p* < 0.05). The most remarkable negative association was presented between PHB2 and plasmacytoid dendritic cells (cor = −0.44, *p* < 0.05). The risk score had a notable positive connection with four types of differential immune cells, and among them, a meaningful positive association was with natural killer T cells and plasmacytoid dendritic cells (cor = 0.38, *p* < 0.05) (Figure 10d).

### 2.7. Immunotherapy and Somatic Mutation in the HRG and LRG 

**Figure 11:**
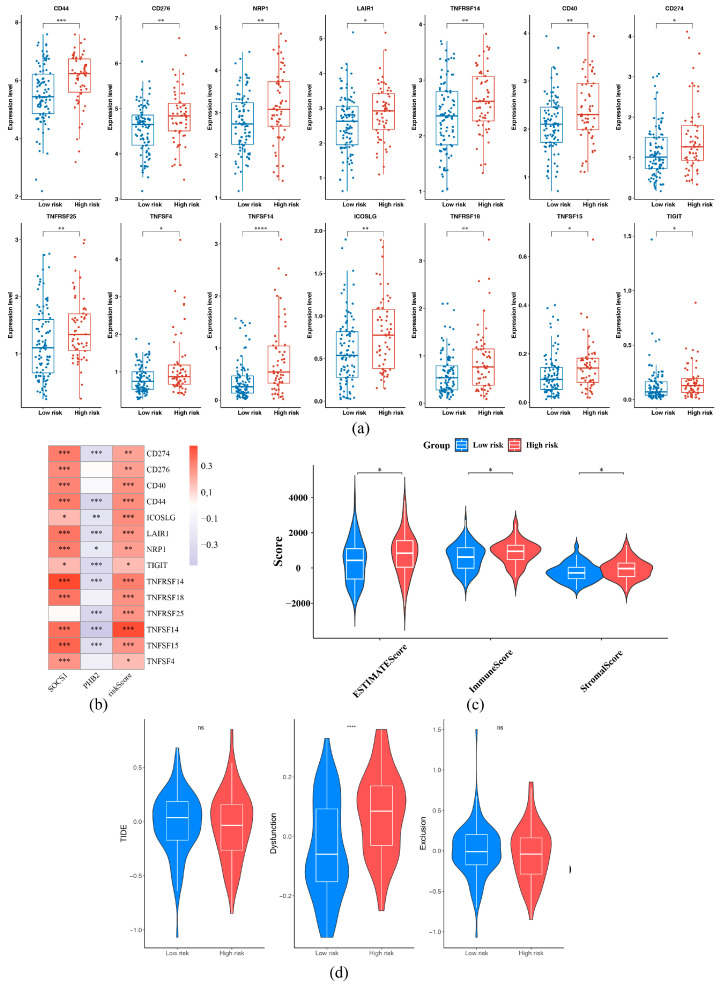
Immune landscape in high and low-risk groups in glioblastoma. (**a**) Expression of immune checkpoints with differences between the high- and low-risk groups, with the high-risk group in red and the low-risk group in blue. Vertical coordinates are immune checkpoint gene expression levels. (**b**) Correlation Analysis Between Prognostic Genes, Risk Scores, and Differential Immune Checkpoints. The larger the positive correlation, the closer the color converges to red. The larger the negative correlation, the more the color converges to blue, and * reflects significance. **** represented *p* < 0.0001, *** represented *p* < 0.001, ** represented *p* < 0.01, * represented *p* < 0.05, and ns represented *p* > 0.05. (**c**) ESTIMATE Analysis. The distribution of Stromal Score, Immune Score, and ESTIMATE Score in the samples of the high- and low-risk groups is presented in the figure. Statistical significance was assessed by the Wilcoxon rank sum test and is shown as a *p*-value in the figure. (**d**) TIDE Analysis. The distribution of TIDE scores and MIS scores in the samples of the high- and low-risk groups is presented in the figure. Statistical significance was assessed by the Wilcoxon rank sum test and is shown as a *p*-value in the figure.

Furthermore, a total of 14 immune checkpoints were found to have notable differences between the HRG and LRG (*p* < 0.05), and all of them were expressed at higher levels in the HRG, such as CD274, LAIR1, TIGIT, TNFSF15, TNFSF4, and CD276 (Figure 11a). The results showed that HRG had more prominent immune escape and a stronger response to immune checkpoint inhibitors. Furthermore, positive interrelations were observed between SOCS1 and nine differential immune checkpoints, among which the most remarkable positive correlation was with TNFRSF14 (cor = 0.45, *p* < 0.05). Negative associations were presented between PHB2 and TNFSF14, CD44, TNFRSF25, and TIGIT, and the most remarkable negative connection among them was with TNFSF14 (cor = −0.46, *p* < 0.05). Positive links were found between risk score and TNFRSF14, TNFSF14, with correlation coefficients being 0.32 and 0.44, respectively (*p* < 0.05) (Figure 11b, Supplementary Table S10). The stromal score, immune score, and ESTIMATE score in the HRG were all substantially more than those in the LRG (*p* < 0.05) (Figure 11c). The Dysfunction score in the HRG was substantially greater than that in the LRG (*p* < 0.05) (Figure 11d). These results indicated that HRG patients were unlikely to benefit from treatment with immunotherapy.

**Figure 12:**
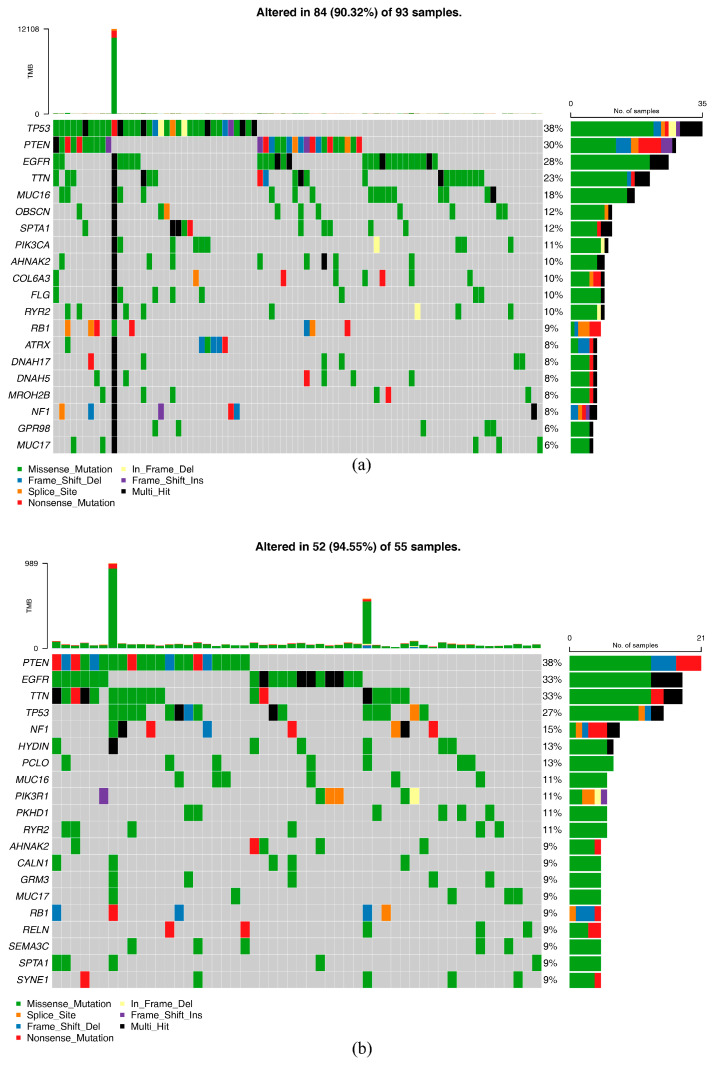
Analysis of tumor mutations in the high-risk and low-risk groups (**a**) Mutation analysis of the high-risk group: for each figure, the middle-left panel depicts the mutation pattern of the gene for each sample, the numbers on the right are the mutation frequency for each gene, the right bar is the proportion of each mutation type for the gene, and the upper bar is the total mutation load for each sample. (**b**) Mutation analysis of the low-risk group.

There were 55 and 93 cases of GBM patients with mutation information in the HRG and LRG, respectively, and the mutation type that occurred most frequently in both groups was missense mutation. Among patients in the HRG, the three genes with the highest number of mutations were PTEN, EGFR, and TTN, with mutation rates of 38%, 33%, and 33%, respectively (Figure 12a). While among patients in the LRG, the three genes with the highest number of mutations were TP53, PTEN, and EGFR, with mutation rates of 38%, 30%, and 28%, respectively (Figure 12b).

### 2.8. Chemotherapeutic Drug Association with Risk Score

**Figure 13:**
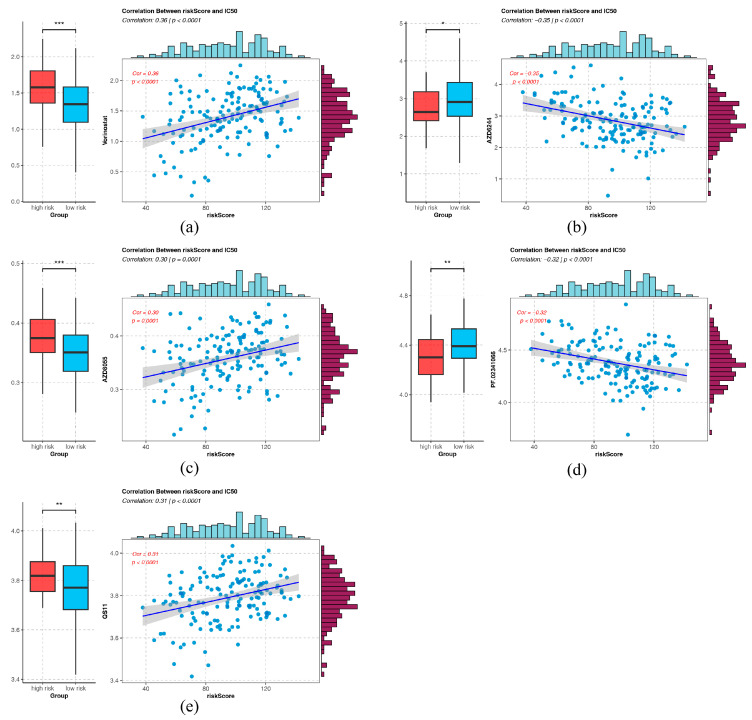
Drug sensitivity analysis in the high and low-risk groups. Each letter (**a**–**e**) represents a different drug ((**a**) Vorinostat; (**b**) AZD6244; (**c**) AZD8055; (**d**) PF.02341066; (**e**) QS11.), and it shows the drug sensitivity analysis based on the IC_50_ values and their correlation with risk scores. The left side highlights the linear relationship between risk scores and IC_50_ values, while the right side compares the IC_50_ values between the high-risk and low-risk groups, showing significant differences in drug sensitivity. *** represented *p* < 0.001, ** represented *p* < 0.01, * represented *p* < 0.05.

Among 138 kinds of chemotherapeutic drugs, IC_50_ values of 41 drugs were found to have notable distinctions (*p* < 0.05) between the HRG and LRG (Supplementary Table S11). Significant associations were observed between IC_50_ values of 5 drugs and risk score. Among them, vorinostat had the most remarkable positive correlation (cor = 0.36, *p* < 0.05) and higher IC_50_ values in the HRG, while AZD6244 had the most remarkable negative correlation (cor = −0.35, *p* < 0.05) and lower IC_50_ values in the HRG (Figure 13a–e). These results indicated that drugs with notable associations might be provided as options for personalized treatment of GBM patients, and the lower IC_50_ values, the more sensitive HRG patients were to chemotherapeutic drugs.

### 2.9. Molecular Regulatory Networks of Prognostic Genes

**Figure 14:**
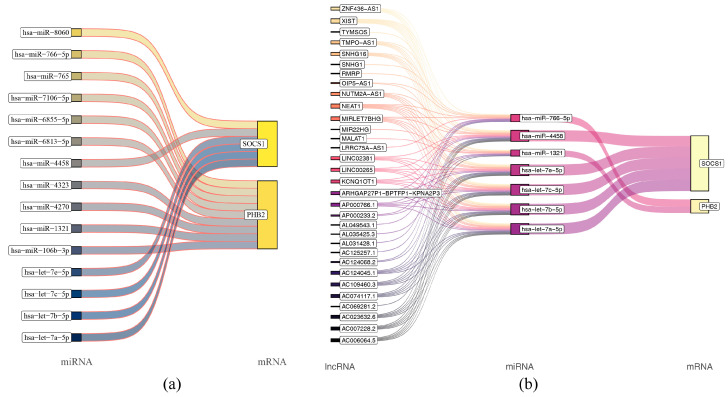
Molecular regulatory networks of prognostic genes. (**a**) Key miRNA-mRNA interaction network. (**b**) lncRNA-miRNA-mRNA interaction network; only key miRNA results that are predictive of lncRNAs are plotted.

PHB2 was regulated by nine key miRNAs, and SOCS1 was adjusted by six miRNAs (Figure 14a). The miRNA-mRNA network had 17 nodes and 15 edges. For example, PHB2 was controlled by hsa-miR-765 and hsa-miR-766-5p, and SOCS1 was regulated by hsa-miR-8060 and hsa-miR-4458 (Figure 14a). There were 49 nodes and 127 edges in the lncRNA-miRNA-mRNA network. For example, PHB2 was directly controlled by hsa-miR766-5p and indirectly adjusted by AC124068.2. SOCS1 was directly controlled by hsa-let-7e-5p indirectly adjusted via AC006064.5 (Figure 14b). Notably, molecular regulatory network analysis revealed that PHB2 and SOCS1 were controlled by multiple miRNAs and lncRNAs.

### 2.10. Cellular Heterogeneity and the Role of Microglia as a Key Cell

**Figure 15:**
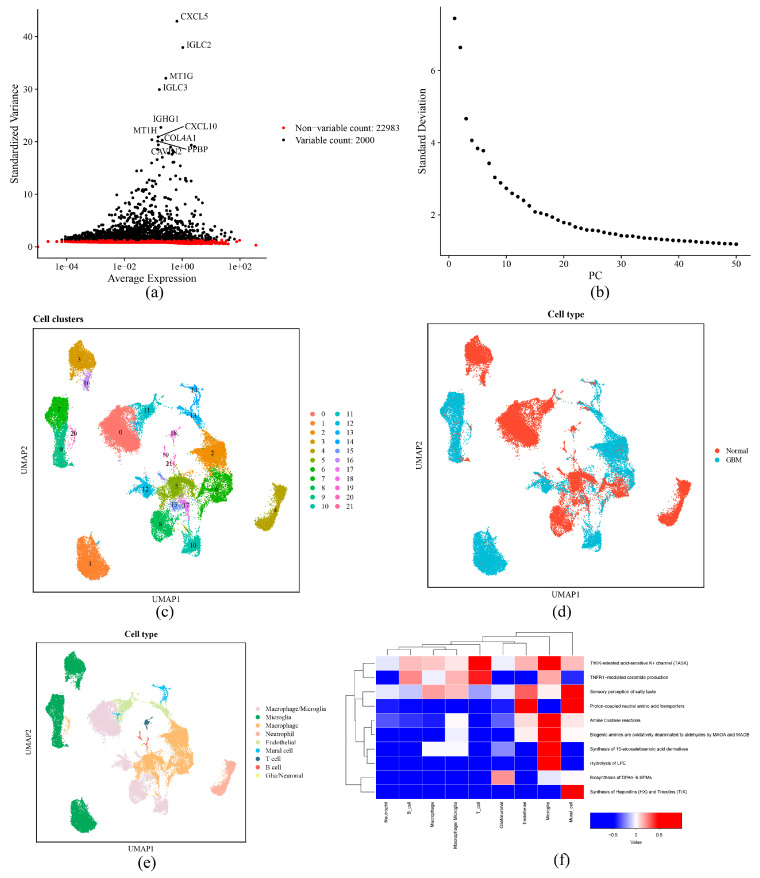
The cellular interactions and functions of microglia. (**a**) Selection plot of highly variable genes. The top 10 highly variable genes are labeled in the graph to highlight their importance in the cell population. (**b**) Confirmed optimal number of clusters. The following figure demonstrates the percentage of cumulative variance for the first 50 principal components. (**c**,**d**) UMAP cell distribution and source classification. (**e**) Cell annotation results. (**f**) Heatmap of GSVA. The heatmap shows the top 10 most differentiated biological pathways identified by GSVA in different cell types.

After quality control of scRNA-seq data, 43,960 cells and 24,983 genes were obtained (Supplementary Figure S1). A total of 2000 highly variable genes were identified (Figure 15a). The PCA results showed that eight cell samples were distributed dispersedly (Supplementary Figure S2a,b). Meanwhile, in PCA, the top 30 principal components were selected for analysis (*p* < 0.05) (Figure 15b). The UMAP clustering analysis identified a total of 22 different cell clusters (resolution = 0.3) (Figure 15c,d). Based on expression of marker genes, cell annotation of different cell clusters was performed, and nine cell types such as macrophage/microglia, microglia, and macrophage were gained (Figure 15e, Supplementary Figure S2c,d).

**Figure 16:**
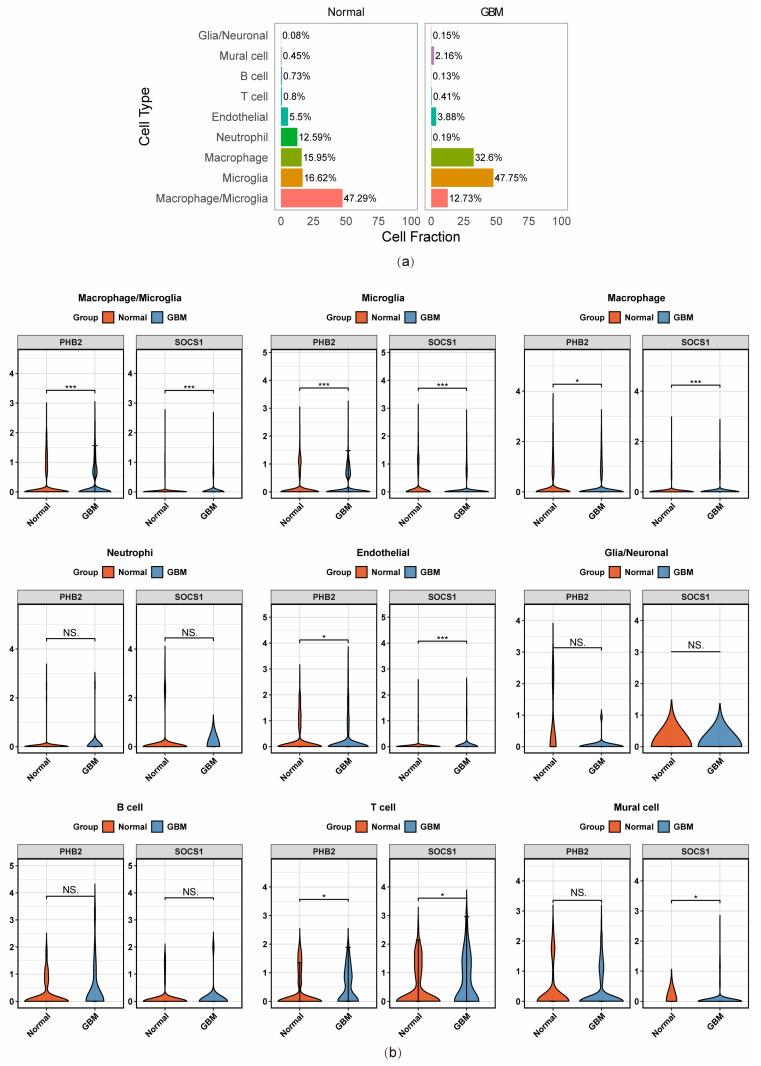
Identification of key cell clusters. (**a**) Plot of cell type proportions. Different colored bars indicate different cell types. (**b**) Violin plots of differences in prognostic gene expression. *** represented *p* < 0.001, * represented *p* < 0.05, and ns represented *p* > 0.05.

Functional enrichment analysis of different cell cluster types yielded 1747 pathways (Supplementary Table S12), such as the synthesis of hepoxilins (HX) and trioxilins (TrX), and synthesis 15-eicosatetraenoic acid derivatives, and biogenic amines are oxidatively deaminated to aldehydes by MAOA and MAOB (Figure 15f). Among different cell types, abundances of microglia and macrophage in tumor tissues were more than those in normal tissues, while neutrophil was greater in normal tissues (Figure 16a). SOCS1 and PHB2 were substantially overproduced in T cells. Meanwhile, in microglia, macrophages, endothelial cells, mural cells, and T cells, there were notable differences in expression levels of prognostic genes (Figure 16b, Supplementary Figure S3). Considering the relationship between microglia and GBM, microglia were identified as key cells.

### 2.11. Cell Communications and Pseudo-Temporal Microglia

**Figure 17:**
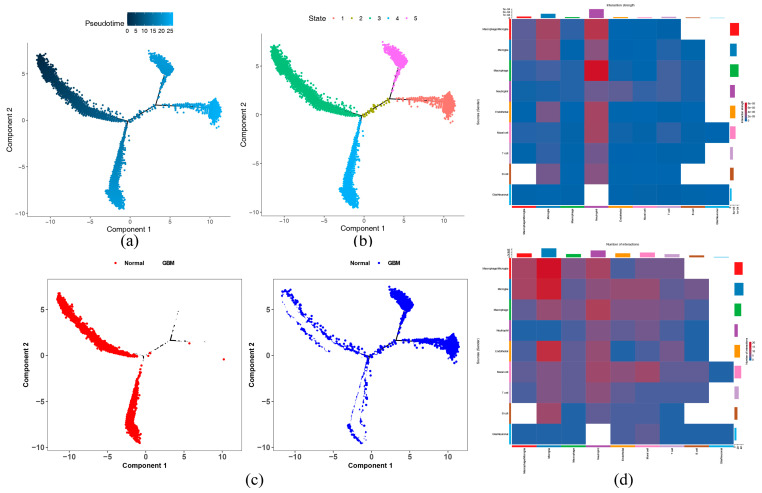
Pseudo-temporal dynamics and communication networks of microglia. (**a**) Simulation analysis of trajectory differentiation. The distribution of cells in pseudo time is indicated by different colors, demonstrating the dynamic changes in cell state. The darker the blue color, the earlier the cells have differentiated. Cells differentiate over time from left to right, with the lightest blue color representing the most recently differentiated cell. (**b**) State Trajectory Analysis. Cell trajectories in different states are identified using colors to identify the different states. (**c**) Population cell type trajectory analysis. The movement trajectories of two groups of cells (normal, tumor) in the pseudo-time dimension are shown. (**d**) Heat map of ligand-receptor pair interactions between key cells. The vertical axis is the cell that sends the signal, the horizontal axis is the cell that receives the signal, and the color depth of the heatmap represents the signal strength. The bars on the top and right are the accumulation of the number of vertical and horizontal axes.

Pseudotime analysis showed that microglia had three differentiation directions and differentiated from left to right over time. The darkest blue indicated earliest differentiated cells, and lightest blue represented most recently differentiated cells (Figure 17a). In addition, microglia exhibited five differentiation states, with 3 being the earliest differentiation type and 1 and 5 being later differentiation types (Figure 17b). Microglia in normal samples were concentrated in differentiation state 3 and differentiation state 4, while microglia in tumor samples were concentrated in differentiation state 1, differentiation state 2, and differentiation state 5 (Figure 17c). As cells differentiated, the expression level of SOCS1 first increased and then decreased, while the expression level of PHB2 increased with cell differentiation (Supplementary Figure S4A). The results of the cell–cell communication network showed that there was communication between microglia and several other cell types (Supplementary Figure S4B). The heatmap of ligand-receptor pairs showed that cell communication between macrophage/microglia and microglia was relatively frequent, and communication intensity between macrophage and neutrophil was relatively high (Figure 17d). The communication signal molecules among various cells indicated that the main signals from other cells to microglia included C3-C3AR1, C3-(ITGAX + ITGB2), etc., and the main signals from microglia to other cells included C3-C3AR1, C3-(ITGAX + ITGB2), etc., among other cells, ILB-IL1R2 had a relatively higher communication signal (Supplementary Figure S4c).

## 3. Discussion

Lipid metabolism plays a crucial role in GBM progression and immune escape, with several LMRGs contributing to tumor survival, metabolic adaptation, and immunosuppression [22]. SREBP1 (Sterol Regulatory Element-Binding Protein 1) and FASN (Fatty Acid Synthase) enhance lipid biosynthesis, promoting tumor proliferation while modulating PD-L1 expression to facilitate immune evasion [28,29]. CD36 (Fatty Acid Translocase) and ACSL4 (Acyl-CoA Synthetase Long-Chain Family Member 4) drive fatty acid uptake and storage, reprogramming the TME through the induction of immunosuppressive M2 macrophages and Tregs [30]. Additionally, CPT1A (Carnitine Palmitoyltransferase 1A)-mediated fatty acid oxidation alters T-cell metabolism, leading to T-cell exhaustion and impaired anti-tumor responses [31]. Tumor cells also manipulate phospholipid remodeling (LPCAT1) and lipid droplet formation (PLIN2) to evade immune detection and sustain growth under metabolic stress [32]. Moreover, LXRα/β signaling reduces antigen presentation, further hindering immune surveillance [33]. These findings highlight the critical role of lipid metabolism in shaping the tumor-immune landscape, and targeting key LMRGs may provide a novel therapeutic approach to disrupt metabolic adaptations, enhance immune responses, and overcome tumor resistance mechanisms in GBM.

The suppressor of cytokine signaling (SOCS) protein family comprises eight members, including SOCS1-SOCS7 and cytokine-inducible SH2-containing protein [34]. SOCS proteins regulate GBM biogenesis primarily via the JAK/STAT and NF-κB signaling pathways, acting as negative regulators to inhibit tumor cell proliferation, invasion, and survival. In addition, SOCS proteins suppress receptor tyrosine kinase (RTK) signaling, a critical pathway involved in tumor cell survival and therapeutic resistance [35]. Dysregulation of RTK signaling, either through overexpression or mutation, contributes to malignant transformation and tumor progression. Beyond their role in tumor cell signaling, SOCS proteins are key modulators of the immune response in GBM. SOCS1 negatively regulates cytokine signaling, including interferons (IFNs) and interleukins (ILs), to prevent excessive immune activation [36]. In the GBM TME, dysregulated SOCS1 and SOCS3 expression suppress anti-tumor immune responses by impairing CD8+ T cell and NK cell function. IL-1β and IL-6, regulated by JAK/STAT and NF-κB signaling, promote tumor proliferation and immune evasion, while IL-10 and TGF-β drive M2 macrophage polarization and Treg expansion, fostering an immunosuppressive tumor milieu. SOCS1-mediated inhibition of JAK/STAT signaling downregulates IL-12, a key activator of cytotoxic T lymphocytes (CTLs) and NK cells, further weakening anti-tumor immunity. Additionally, SOCS3 interferes with IFN-γ-mediated responses, reducing MHC class I antigen presentation and impairing T cell-mediated tumor recognition. Furthermore, SOCS-driven modulation of PD-L1 expression may exacerbate immune escape mechanisms, further attenuating immune surveillance in GBM [37]. These findings further validate the reliability of our results and underscore the pivotal role of SOCS1 in GBM pathogenesis.

PHB2 (Prohibitin 2) is an evolutionarily conserved protein primarily localized in mitochondria and the nucleus [37]. In mitochondria, PHB2 forms a complex with PHB1, which is essential for maintaining mitochondrial structural integrity. PHB2, along with PHB1, belongs to the Prohibitin (PHB) protein family and is associated with cellular aging, influencing aging-related signaling pathways through changes in expression levels and subcellular localization [38]. In glioma stem-like cells (GSCs), PHB2 specifically regulates mitochondrial ROS production, contributing to radiotherapy resistance. Dysregulated ROS levels can enhance DNA damage repair mechanisms, reduce oxidative stress-induced apoptosis, and promote tumor cell survival, leading to increased resistance to radiotherapy. Mechanistically, PHB2 interacts with mitochondrial electron transport chain (ETC) complexes to modulate ROS production, thereby influencing redox balance and metabolic adaptation in GBM. Increased PHB2 expression has been associated with enhanced mitochondrial bioenergetics, providing a survival advantage under radiotherapy-induced oxidative stress [39]. In addition to its role in ROS regulation, PHB2 influences cell cycle progression by modulating cyclin expression. Specifically, PHB2 upregulates Cyclin D1 and Cyclin E, facilitating G1/S phase transition and promoting uncontrolled tumor cell proliferation. Furthermore, PHB2 has been shown to increase Cyclin A and Cyclin B expression, accelerating S/G2 phase progression and mitotic entry, thereby supporting rapid tumor growth and therapy resistance. Conversely, PHB2 inhibits cyclin-dependent kinase inhibitors (CKIs), such as p21 and p27, further enhancing cell cycle progression and reducing tumor cell susceptibility to DNA damage-induced apoptosis [40,41]. Given these multifaceted roles, PHB2 is emerging as a potential therapeutic target for GBM.

Current standard chemotherapy for GBM primarily relies on temozolomide (TMZ) in combination with radiotherapy, yet its efficacy is often limited by the development of resistance mechanisms, such as MGMT promoter methylation status and DNA repair pathway activation [42]. Despite these interventions, tumor recurrence and therapy resistance remain significant challenges, underscoring the need for novel therapeutic strategies. Our study identifies SOCS1 and PHB2 as key genes implicated in CS and lipid metabolism, which may play a critical role in GBM progression and treatment response. Given that SOCS1 downregulation enhances PD-L1 expression and NF-κB activation, it may contribute to immune evasion and reduced sensitivity to immunotherapy or chemotherapy. Meanwhile, PHB2 has been linked to radiotherapy resistance. Given its role in cell cycle regulation (Cyclin D1, Cyclin E) and mitochondrial stress responses, PHB2 may influence the efficacy of TMZ and other chemotherapeutic agents by modulating tumor metabolism and oxidative stress responses. Our findings suggest that targeting SOCS1 and PHB2 may enhance the efficacy of existing GBM therapies. Future research should focus on validating these findings in patient-derived GBM models and exploring potential targeted therapies or combination treatment strategies. The integration of genetic profiling and personalized therapy approaches could optimize GBM treatment outcomes, ultimately translating these molecular insights into clinically actionable therapeutic interventions.

Previous research has demonstrated that hsa-miR-765 regulates cancer progression by modulating the ERK/Akt/AMPK signaling pathway and the expression of HMGA1 protein [43]. In prostate cancer, hsa-miR-765 inhibits cell proliferation, migration, and invasion, suggesting a potentially similar role in GBM [44]. Meanwhile, hsa-miR-766-5p has been implicated in critical cellular processes, such as apoptosis and differentiation, and is recognized as a novel regulator of the mitochondrial apoptosis pathway [45]. However, its specific role in GBM remains underexplored. In this study, regulatory network analysis revealed that PHB2 and SOCS1 are modulated by multiple miRNAs and lncRNAs. Specifically, hsa-miR-765 and hsa-miR-766-5p directly target PHB2, while SOCS1 is regulated by hsa-miR-8060 and hsa-miR-4458. Additionally, lncRNAs indirectly influence the expression of these genes through the ceRNA mechanism. hsa-miR-765 and hsa-miR-766-5p target PHB2, leading to post-transcriptional inhibition, which disrupts mitochondrial function and ROS production, potentially enhancing radiotherapy sensitivity. Meanwhile, hsa-miR-8060 and hsa-miR-4458 suppress SOCS1, affecting JAK/STAT and NF-κB signaling, thereby promoting tumor proliferation and immune evasion. PHB2 regulates cell cycle progression by modulating cyclin expression: its upregulation enhances Cyclin D1 and Cyclin E (G1/S transition) and Cyclin A and Cyclin B (S/G2 progression), driving tumor growth [46]. Conversely, miRNA-induced PHB2 inhibition reduces Cyclin D1 and Cyclin A, leading to cell cycle arrest and increased therapy sensitivity. Similarly, SOCS1 downregulation via miRNAs sustains STAT3 activation, resulting in Cyclin D1/E upregulation (G1/S transition), PD-L1-mediated immune evasion, and NF-κB-driven survival signaling [47]. In conclusion, miRNA-mediated suppression of PHB2 and SOCS1 contributes to cell cycle dysregulation, radiotherapy resistance, and immune escape in GBM, highlighting their potential as therapeutic targets.

Among the most significantly dysregulated pathways in GBM, the PI3K/Akt/mTOR and MAPK/ERK pathways play a central role in tumor progression [48,49]. PTEN, a key negative regulator of PI3K signaling, is frequently mutated or deleted in GBM, leading to uncontrolled cell growth and survival [50]. Additionally, EGFR amplifications and mutations drive MAPK and PI3K/Akt hyperactivation, promoting tumor proliferation and resistance to apoptosis [51]. Furthermore, pathway enrichment analysis has identified several signaling pathways as key contributors to GBM progression. Among these, the JAK-STAT signaling pathway plays a pivotal role in tumorigenesis, regulating immune responses, cell proliferation, and tumor progression, thereby influencing the complex interplay between the GBM microenvironment and tumor cell survival [52]. Similarly, the PP2A-AKT signaling pathway is critical for lipid metabolism and cellular proliferation, affecting GBM metabolic reprogramming and therapeutic resistance [53]. The involvement of these pathways underscores the complexity of GBM regulation and their potential as therapeutic targets, emphasizing the need for further investigation into their roles in CS and lipid metabolism in GBM.

The risk model constructed using two prognostic genes, SOCS1 and PHB2, demonstrated strong predictive power for GBM prognosis. Validated across TCGA-GBM and independent datasets, the model stratified patients into high-risk and low-risk groups using optimal cutoff values (103.013990 in TCGA-GBM; 87.568442 in validation). High-risk patients had significantly lower survival probabilities (*p* < 0.05), as shown by K-M curves and ROC analysis, with AUC values of 0.746, 0.737, and 0.831 for 1-, 2-, and 3-year survival in TCGA, and 0.843, 0.698, and 0.811 in the validation set, confirming its stability and reproducibility. Clinicopathological analysis revealed poor survival outcomes in high-risk patients across subgroups defined by age, sex, IDH1 mutation, and MGMT methylation status. Univariate and multivariate Cox regression confirmed the risk score as an independent prognostic factor (*p* < 0.05). A nomogram incorporating the risk score, IDH1 mutation, and MGMT methylation predicted 1-, 2-, and 3-year survival with AUC values of 0.75, 0.77, and 0.84. While the prognostic performance of our model may not be the most outstanding compared to other related prognostic models, it still outperforms the majority of reported GBM prognostic models [54,55,56].

In this study, missense mutations were the most prevalent type of somatic mutation in both the HRG and LRG. Among HRG patients, the top three mutated genes were PTEN (38%), EGFR (33%), and TTN (33%), whereas in LRG patients, the most frequently mutated genes were TP53 (38%), PTEN (30%), and EGFR (28%). These findings align partially with prior large-scale genomic studies, which also identified PTEN [57], TP53 [58], and EGFR [59] as hotspot genes in GBM, emphasizing their central role in tumor progression. However, the mutation frequency of TTN in the HRG appears higher than typically reported [60], suggesting potential group-specific genetic vulnerabilities. Our analysis identified vorinostat and AZD6244 as potential candidates for stratified therapeutic approaches in GBM. Vorinostat, a histone deacetylase inhibitor [61], appears less effective in the HRG due to higher IC_50_ values, making it more suitable for LRG patients. In contrast, AZD6244, a MEK inhibitor [62], demonstrated efficacy in HRG patients by potentially targeting aberrant MAPK signaling pathways, which are often upregulated in aggressive GBM phenotypes. Other drugs, including AZD8055 and PF.02341066, also showed significant IC_50_ differences, underscoring their potential utility in risk-stratified GBM treatments. The observed differences in drug sensitivities between the HRG and LRG emphasize the importance of integrating molecular profiling into GBM therapy planning. Additionally, these findings support further research into the clinical and preclinical evaluation of drug efficacy, especially for agents demonstrating differential responses across GBM subgroups.

Microglia play a crucial role in GBM progression, tumor heterogeneity, and immune modulation by interacting with tumor cells and shaping the TME [63]. Key microglia-associated genes, including TREM2 and CX3CR1, regulate immune suppression, tumor proliferation, and invasion, while IL-1β and TNF-α contribute to tumor-promoting inflammation [64]. Conversely, anti-inflammatory mediators such as IL-10 and TGF-β drive immunosuppressive (M2-like) microglial polarization, facilitating immune evasion [65]. Additionally, CSF1R is critical for microglial survival and proliferation, making it a potential therapeutic target to reduce microglia-mediated tumor promotion [66]. These findings highlight the pivotal role of microglia in GBM pathogenesis, suggesting that targeting TREM2, CX3CR1, and CSF1R may offer novel strategies to counteract microglia-driven immune suppression and enhance anti-tumor immunity.

Single-cell RNA sequencing provides a high-resolution approach to identifying diverse cell types within GBM tissues. In this study, cell annotation identified nine cell types, with macrophages and microglia significantly enriched in tumor tissues compared to normal tissues, indicating their critical role in the TME. Conversely, neutrophils exhibited higher abundance in normal tissues, highlighting distinct immune dynamics between tumor and non-tumor environments. The proportion of tumor-associated macrophages (TAMs) increased significantly during GBM progression, suggesting their role in promoting tumor growth and invasion. These findings align with previous reports highlighting TAMs as key contributors to the immunosuppressive TME and GBM aggressiveness [67].

Prognostic genes SOCS1 and PHB2 identified in this study exhibited dynamic expression patterns in microglia. SOCS1 expression initially increased and then decreased during microglial differentiation, while PHB2 expression progressively increased. This temporal regulation suggests distinct roles for these genes in GBM progression, with SOCS1 likely contributing to early-stage immune modulation and PHB2 supporting later-stage metabolic and survival adaptations in microglia. The enrichment of microglia in the GBM microenvironment and their active communication with other cell types highlight their dual role in modulating immune responses and supporting tumor progression. Targeting microglia-specific pathways, such as the SOCS1/PHB2 expression, may represent promising strategies for developing effective GBM therapies.

This study provides a comprehensive bioinformatics analysis of publicly available databases to explore the genetic diversity of GBM. By integrating multi-omics datasets, we identified potential prognostic genes and therapeutic targets. Our results highlight the predictive power of bioinformatics in identifying key molecular signatures associated with GBM prognosis and therapeutic resistance. The integration of large-scale datasets allows for unbiased and systematic biomarker discovery, providing a valuable foundation for future clinical validation.

Despite the significant findings presented in this study, several limitations must be acknowledged. First, the conclusions rely predominantly on bioinformatic analyses of publicly available datasets, which may not fully reflect the heterogeneity of GBM across diverse patient populations. To bridge the gap between computational predictions and clinical applications, future research should focus on prospective clinical studies and experimental validation using patient-derived samples. Second, the roles of SOCS1 and PHB2 in GBM progression remain speculative, requiring in-depth experimental validation to elucidate their underlying mechanisms. Moving forward, it will be essential to validate these bioinformatically predicted biomarkers using patient tumor tissues, functional assays, and preclinical models. Third, clinical validation with larger, independent patient cohorts is necessary to confirm the generalizability and clinical utility of the proposed risk model. Looking ahead, we plan to further investigate the biological mechanisms underlying these pathways and genes, with the ultimate goal of translating these findings into clinical practice. By addressing these limitations, our study lays the foundation for a deeper understanding of GBM and paves the way for the development of more effective and personalized therapeutic strategies.

## 4. Materials and Methods

### 4.1. Data Harvesting

The bulk RNA sequencing (RNA-seq) dataset (the Cancer Genome Atlas-Glioblastoma (TCGA-GBM)) was obtained from University of California Santa Cruz (UCSC) Xena (https://xena.ucsc.edu/ (accessed on 24 September 2024)) on 24 September 2024, which encompassed 154 GBM samples with survival information (Supplementary Table S13). Subsequently, two microarray datasets (GSE68848 (GPL570) and GSE74187 (GPL6480)) and a single-cell RNA-seq (scRNA-seq) dataset (GSE162631 (GPL24676)) were obtained from Gene Expression Omnibus (GEO) database (https://www.ncbi.nlm.nih.gov/geo/ (accessed on 24 September 2024)). The GSE68848 dataset was downloaded on 25 September 2024, served as a training set, and comprised 228 GBM samples and 28 normal samples, all of which were brain tissue samples. GSE74187 dataset was downloaded on 24 September 2024, functioned as validation set and included 60 GBM samples with follow-up time and death information. The GSE162631 dataset was downloaded on 29 September 2024, containing 4 GBM tissue samples and 4 adjacent normal tissue samples. Additionally, 866 CSAGs were downloaded from the CellAge database (https://genomics.senescence.info/cells/ (accessed on 25 September 2024)) (Supplementary Table S15). Moreover, 17 LMRG sets were extracted from the Molecular Signatures Database (MSigDB) (https://www.gsea-msigdb.org/gsea/msigdb (accessed on 25 September 2024)), encompassing 1070 LMRGs. Concurrently, 15 lipid LMRG sets were gained from the Kyoto Encyclopedia of Genes and Genomes (KEGG) database (https://www.genome.jp/kegg/ (accessed on 25 September 2024)), which included 654 LMRGs. After merging and eliminating duplicate genes, a total of 1133 LMRGs were finally obtained (Supplementary Table S14).

### 4.2. Weighted Gene Co-Expression Network Analysis (WGCNA)

In the training set, WGCNA was accomplished on expression matrices of all samples through the WGCNA (v 1.71) package [68]. First, hierarchical clustering was carried out by the Euclidean distance of expression levels to check whether there were outliers in samples, and outlier samples were removed. Subsequently, the critical value when R^2^ exceeded 0.85 for the first time and average connectivity of co-expression network was close to 0 were utilized to obtain optimal soft thresholding and envisioned by plot function. Founded on optimal soft thresholding, a scale-free network was established. The minimum number of genes in each module was set at 100, with mergeCutHeight set at 0.25, enabling the genes to be effectively grouped into multiple modules, envisioned by the plotDendroAndColors function. Then, the grouping information of GBM and normal samples was regarded as the phenotype. The cor function was used for Pearson correlation analysis between the modules and phenotype (|correlation (cor)| > 0.3, *p* < 0.05). The labeled Heatmap function was applied to draw an association heatmap to display results. The markedly correlated modules were selected, and the verboseScatterplot function was applied to draw a scatter plot between Gene Significance (GS) and Module Membership (MM) (|MM| > 0.6, |GS| > 0.2) to further screen genes in notable modules [69]. The genes gained from each module were combined to gain WGCNA-related genes.

### 4.3. Recognition of Candidate Genes

In the training set, differential analysis was performed between GBM and normal samples via the limma (v 3.54.0) package [70] to gain differentially expressed genes (DEGs) (|log2 Fold Change (FC) | > 0.5, *p* < 0.05). The ggplot2 (v 3.4.4) package [71] was applied to display a volcano plot and a circular heatmap to visualize DEGs. The top 10 up and downregulated genes ranked according to log2FC were labeled in the volcano plot, and the genes presented in the heatmap corresponded to them. Finally, the UpSetR (v 1.4.0) package [72] was used to analyze WGCNA-related genes, DEGs, CSAGs, and LMRGs to ascertain intersection genes as candidate genes, which were visualized by an UpSet plot.

### 4.4. Enrichment Analysis of Candidate Genes and Construction of Protein–Protein Interaction (PPI) Network

To explore biological functions and signal pathways of candidate genes, the clusterProfiler (v 4.7.1.003) [73] and org.Hs.eg.db (v 3.16.0) packages [74] were used to perform Gene Ontology (GO) and KEGG enrichment analysis (*p* < 0.05). The GO enrichment analysis consisted of three sections: biological processes (BPs), cellular components (CCs), and molecular functions (MFs). The results were ranked in ascending order in accordance with the *p*-value. For three parts of GO results, the top 5 results were, respectively, selected, and top 10 KEGG results were selected for display, depicted through the ggplot2 (v 3.4.4) package. Subsequently, to investigate protein-level interactions of candidate genes, they were sent to the Search Tool for the Retrieval of Interacting Genes/Proteins (STRING) database (https://string-db.org/ (accessed on 25 September 2024)) for PPI analysis. (combined score ≥ 0.15). After removing isolated genes, the results were illustrated using Cytoscape (v 3.9.1) software [75].

### 4.5. Construction and Validation of Risk Model

In the TCGA-GBM dataset, univariate Cox regression analysis was executed on candidate genes by survival (v 3.5-3) package [76] (*p* < 0.05). Subsequently, a proportional hazards (PH) assumption test was carried out for each gene (*p* > 0.05) to acquire prognostic genes. The Random Survival Forest (RSF) model [77] was formed for prognostic genes via randomForestSRC (v 3.2.2) package (http://cran.r-project.org/web/packages/randomForestSRC/index.html (accessed on 25 September 2024)) to predict risk score of each patient (ntree = 11, mtry = 1). The formula was as follows:

$$h\left(t|x\right)=\frac{1}{B}\sum_{i=1}^{B}h_{i}\left(t|x\right)$$

The $h\left(t|x\right)$ was prediction result of ith tree for individual *x* at time *t*. The GBM samples were divided into a high-risk group (HRG) and a low-risk group (LRG) in line with the optimal cut-off value of the risk score. The ggplot2 (v 3.4.4) and gridExtra (v 2.3) packages (https://cran.r-project.org/web/packages/gridExtra/index.html (accessed on 25 September 2024)) were used to draw the risk score distribution plot and survival status distribution plot, respectively. The survival package (v 3.5-3) was utilized to compare the overall survival time (OS.time) and survival status (OS) ranging from the HRG to the LRG, which were presented using a Kaplan–Meier (K-M) survival curve (*p* < 0.05). The survivalROC (v 1.0.3.1) package [78] was utilized to draw receiver operating characteristic (ROC) curves for 1 year, 2 years, and 3 years (area under the curve (AUC) > 0.7) to evaluate the prediction effectiveness of the risk model in GBM samples. The ComplexHeatmap (v 2.14.0) package [79] was used to draw a heatmap to display expression distinctions of prognostic genes in the HRG and LRG. Meanwhile, the risk model was validated in the validation set in line with the aforementioned methods.

### 4.6. Analysis of Clinicopathological Features

In TCGA-GBM dataset, to investigate distinctions in clinicopathological features among the HRG and LRG, age, gender, isocitrate dehydrogenase1 (IDH1) status, methylguanine methyltransferase (MGMT) promoter status [80], and the expression of prognostic genes in groups were analyzed. A heatmap was drawn via the ComplexHeatmap (v 2.14.0) package for visualization. Subsequently, GBM samples were divided into distinct subgroups pursuant to various clinicopathological traits (age (≤60 years and >60 years), gender (male and female), IDH1 status (mutant and wild type (WT)), MGMT methylation (methylated and unmethylated), and unknown information (unknown)). Firstly, risk status distribution of various clinicopathological feature subgroups was analyzed between two risk groups using the chisq.test function in the stats (v 4.2.2) package [81] (*p* < 0.05). Subsequently, the Wilcoxon test in the ggsignif (v 0.6.4) package (https://cran.r-project.org/web/packages/ggsignif/vignettes/intro.html (accessed on 25 September 2024)) was utilized to contrast risk score differences among disparate subgroups (*p* < 0.05), visualized through the ggplot2 (v 3.4.4) package. Finally, the survival (v 3.5-3) package was utilized to analyze survival variances of the HRG and LRG per subgroup (*p* < 0.05), and a K-M survival curve was drawn. Patients with unknown information were not included in the analysis. Since the number of patients with wild-type IDH1 was relatively small, they were not included in the analysis.

### 4.7. Nomogram Creation and Validation

In the TCGA-GBM dataset, univariate Cox regression analysis was performed on risk score and disparate clinicopathological traits via the survival (v 3.5-3) package (*p* < 0.05). Then, the PH assumption test was carried out (*p* > 0.05). Subsequently, independent prognostic factors were gained through multivariate Cox regression analysis through the survival (v 3.5-3) package (*p* < 0.05). A forest plot was drawn using the forestplot (v 3.1.1) [82] package for visualization. The nomogram was formed for independent prognostic traits via the rms (v 6.5-0) package [83] to predict the 1-year, 2-year, and 3-year survival probabilities of GBM patients. The calibration curve was established through the calibrate function in the rms (v 6.5-0) package to evaluate the prediction accuracy of the nomogram model. ROC curves for 1 year, 2 years, and 3 years were exhibited using the survivalROC (v 1.0.3.1) package [78] to assess the effectiveness of the model (AUC > 0.07). Finally, the decision curve analysis (DCA) curve was exhibited via the ggDCA (v 1.2) package (https://www.rdocumentation.org/packages/ggDCA/versions/1.1 (accessed on 25 September 2024)) to further evaluate the clinical utility of nomogram.

### 4.8. Functional Enrichment Analysis of the HRG and LRG 

In the TCGA-GBM dataset, differential analysis among the HRG and LRG was performed using the DESeq2 (v 1.38.0) package [84] to obtain DEGs, and log2FC was ranked in descending order. The c2.cp.kegg_medicus.v2023.2.Hs.symbols.gmt was downloaded from the MSigDB database as a background reference gene set. Then, the Gene Set Enrichment Analysis (GSEA) function in the clusterProfiler (v 4.7.1.003) package was applied to carry out enrichment analysis on DEGs between two risk groups (|normalized enrichment score (NES) | > 1, *p* < 0.05). Subsequently, the top 5 pathways with smallest *p*-values were selected and depicted via the enrichplot (v 1.18.0) package [85]. Gene Set Variation Analysis (GSVA) was accomplished among the HRG and LRG via the GSVA (v 1.46.0) package [86] to compute enrichment scores of each signal pathway. The background reference gene set was the same as that of GSEA. Then, the limma (v 3.54.0) package was used to contrast pathway differences ranging from the HRG to the LRG (*p* < 0.05, |t| > 2). A t value greater than 2 represented a pathway markedly upregulated in the HRG, and a t value less than 2 represented a pathway substantially downregulated in the HRG. Subsequently, the top 10 pathways with the largest |t| values were selected and presented via the ggplot2 (v 3.4.4) package.

### 4.9. Immune Microenvironment Analysis

In the TCGA-GBM dataset, infiltration analysis of 28 immune cells [87] was carried out by applying the single-sample gene set enrichment analysis (ssGSEA) algorithm in the GSVA (v 1.46.0) package [88], and then the immune scores of each immune cell in the HRG and LRG were obtained. Subsequently, the Wilcoxon test was performed by wilcox.test function in stats (v 4.2.2) package to contrast immune scores of each immune cell between groups (*p* < 0.05), and thus differentially expressed immune cells were identified. Furthermore, the Wilcoxon test method in the rstatix (v 0.7.2) package (https://cran.r-project.org/web/packages/rstatix/index.html (accessed on 25 September 2024)) was utilized to contrast expression levels of 48 immune checkpoints [89] ranging from the HRG to the LRG (*p* < 0.05), and differentially expressed immune checkpoints were acquired. Moreover, box plots were drawn using the ggplot2 (v 3.4.4) package for visualization. In addition, the Spearman method within the cor function of the stats (v 4.2.2) package was used to analyze connections among differentially expressed immune cells and prognostic genes, along with the risk score and differentially expressed immune cells (or differentially expressed immune checkpoints) (|cor| > 0.3, *p* < 0.05). Finally, an association heatmap and bubble plot were, respectively, drawn via the ggcorrplot (v 0.1.4) [90] and ggplot2 (v 3.4.4) packages for visualization.

The Estimation of STromal and Immune cells in MAlignant Tumor tissues through Expression data (ESTIMATE) analysis was conducted using the estimate (v 1.0.13) package (https://bioinformatics.mdanderson.org/estimate/rpackage.html (accessed on 25 September 2024)), and the immune score, stromal score, and ESTIMATE score of each sample were gained. Subsequently, the Tumor Immune Dysfunction and Exclusion (TIDE) dysfunction and exclusion scores were obtained on the TIDE website (http://tide.dfci.harvard.edu/ (accessed on 15 October 2024)). Moreover, the Wilcoxon test was carried out by means of the ggpubr (v 0.6.0) package [91] to analyze distinctions in each score among the HRG and LRG (*p* < 0.05). The violin plots were drawn utilizing the ggplot2 (v 3.4.4) package for visualization.

Furthermore, somatic mutation data of GBM patients were gained from the TCGA database. Then, mutation data of patients in the HRG and LRG were analyzed by the maftools (v 2.26.0) package [92]. After that, genes with the top 20 mutation frequencies were selected for presentation.

### 4.10. Chemotherapeutic Drug Sensitivity

In order to recognize potential drugs for treating GBM, in the TCGA-GBM dataset, 138 kinds of drugs were obtained from the Genomics of Drug Sensitivity in Cancer (GDSC) database (https://www.cancerrxgene.org/ (accessed on 10 October 2024)). Then, half-maximal inhibitory concentrations (IC_50_) values of these drugs for each sample were evaluated using the pRRophetic (v 0.5) package [93] to assess the sensitivity of samples to drugs. After that, discrepancies in IC_50_ values of each drug between the HRG and LRG were compared using the wilcox.test function in the rstatix (v 0.7.2) package (*p* < 0.05). Subsequently, the connection between IC_50_ values and risk scores was calculated by employing the cor.test function in the stats (v 4.2.2) package (|cor| > 0.3, *p* < 0.05). Finally, scatter plots and box plots were drawn by means of the ggplot2 (v 3.4.4) package for visualization.

### 4.11. Construction of Molecular Regulatory Networks

In order to analyze the molecular regulatory mechanism of prognostic genes, the miRWalk database (http://mirwalk.umm.uni-heidelberg.de (accessed on 10 October 2024)) and the experimentally validated microRNA-target interactions database (miRTarBase) (https://mirtarbase.cuhk.edu.cn/~miRTarBase/miRTarBase_2019/php/index.php (accessed on 10 October 2024)) were utilized to predict the microRNA (miRNA) that interacted with the prognostic genes (mRNA). Subsequently, key miRNAs were obtained by taking the intersection of prediction results from two databases using the ggvenn (v 0.1.9) package [94]. After that, the starBase database (https://rnasysu.com/encori/ (accessed on 10 October 2024)) was used to predict the long non-coding RNA (lncRNA) that interacted with key miRNAs. Finally, Cytoscape (v 3.9.1) software was used to draw miRNA–mRNA and lncRNA–miRNA–RNA interaction networks.

### 4.12. scRNA-Seq Data Processing

The 10x scRNA-seq data of GSE162631 was processed via the Seurat (v 5.0.1) package [95]. Firstly, quality control was performed on scRNA-seq data. Cells with fewer than 200 genes and genes covering fewer than 3 cells were screened out. The range of nFeature was set between 400 and 5000, proportion of percent. Mt was less than 5%, and range of nCount_RNA was within 17,000. The results were depicted using the VlnPlot function. Subsequently, the expression data of each cell was normalized using the NormalizeData function, with the scale factor set at 10,000. Then, 2000 highly variable genes were identified by applying the FindVariableFeatures function, and through the VariableFeaturePlot function, they were visualized. After that, dimensionality reduction and clustering were performed on the scRNA-seq data. Principal component analysis (PCA) was carried out via the RunPCA function, and the first 50 PCs were calculated (*p* < 0.05). A scatter plot was formed for visualization using the DimPlot function. Then, the JackStraw function was applied to calculate contributions of the first 50 PCs in order to determine the optimal number of clusters and they were illustrated via JackStrawPlot. Meanwhile, ElbowPlot was used to evaluate the cumulative contributions of PCs to overall data variation to determine the appropriate number of PCs. The adjacency graph of cells was established through the FindNeighbors function, and these cells were clustered using the FindClusters function (resolution = 0.3) to recognize distinct cell types, illustrated using the DimPlot function. Finally, marker genes were obtained from literature [96] to conduct cell annotation for different cell clusters. The expression of marker genes in the annotated cells was plotted via the ggplot2 (v 3.4.4) package, and results of cell annotation were illustrated using the DimPlot function.

### 4.13. Enrichment Analysis of Cells

The analyse_sc_clusters function in the ReactomeGSA (v 1.12.0) package [97] was used to conduct functional enrichment analysis on different types of cell clusters. Then, expression profiles of pathways were extracted through pathways function, and difference values (maximum enrichment score minus minimum enrichment score) were obtained by calculating minimum and maximum enrichment scores of each pathway in diverse cell types. Subsequently, the top 10 pathways with the largest difference values were selected, and a heatmap was drawn for visualization via the plot_gsva_heatmap function.

### 4.14. Identification of Key Cells

The proportions of each cell cluster were calculated, respectively, in GBM samples and normal samples and then illustrated using the ggplot2 (v 3.4.4) package. Subsequently, in order to detect key cells, the overall expression of prognostic genes in all cells was plotted using the FeaturePlot function in the Seurat (v 5.0.1) package, and results were presented using violin plots through the VlnPlot function in the Seurat (v 5.0.1) package. After that, in each cell cluster, respectively, variances in the expression levels of the prognostic genes among the GBM and normal samples were compared (*p* < 0.05) and illustrated via the ggplot2 (v 3.4.4) package. Finally, the cells in which there were differences in the expression levels of the prognostic genes and which were related to GBM were selected as key cells.

### 4.15. Cell Communication and Pseudotime Analysis

Cell communication analysis was conducted on annotated cell clusters using the CellChat (v 1.6.1) package [98]. Then, network diagrams and heatmaps were drawn utilizing the netVisual_heatmap function in the CellChat (v 1.6.1) package to display the results. Furthermore, in order to understand the cell developmental trajectory of key cells and changes in the expression of prognostic genes within cells, cell pseudotime trajectory analysis was performed on key cells using the monocle (v 2.26.0) package [99]. Subsequently, visualization was carried out using the plot_cell_trajectory function.

## 5. Conclusions

In conclusion, our study identified SOCS1 and PHB2 as significant prognostic genes. The risk model and nomogram we constructed demonstrated excellent predictive abilities. Patients in the high-risk group (HRG) exhibited a lower survival probability across various clinicopathological subgroups. We identified a total of 12 differential immune cells and 14 immune checkpoints in both risk groups, with a more pronounced presence in the HRG. The stromal, immune, estimate, and dysfunction scores were significantly higher in the HRG compared to the low-risk group (LRG). Mutation rates for PTEN, EGFR, and TTN were highest in the HRG, while in the LRG, they were highests for TP53, PTEN, and EGFR. The half-maximal inhibitory concentrations (IC50) values of 41 drugs showed significant differences between the two risk groups, indicating potential differences in drug sensitivity. Finally, during the differentiation of key cells (microglia), SOCS1 expression initially increased and then decreased, while PHB2 expression consistently increased. Our findings provided a novel theoretical foundation for the prevention and treatment of GBM, offering new strategies for disease mechanisms, prognostic evaluation, and the development of treatment options.

## Data Availability

The original contributions presented in this study are included in this article and in the Supplementary Materials.

## Supplementary Materials

The following supporting information can be downloaded at: https://www.mdpi.com/article/10.3390/ijms26051875/s1.
