# Supplementary material for: Identification of Prognostic Genes Related to Cell Senescence and Lipid Metabolism in Glioblastoma Based on Transcriptome and Single-Cell RNA-Seq Data
Source: Int J Mol Sci. 2025 Feb 21;26(5):1875. doi: 10.3390/ijms26051875 (PMC11899969; doi:10.3390/ijms26051875)
Supplement: Supplementary file 1 [file ijms-26-01875-s001.zip › ijms-3467288 suppl.pdf]

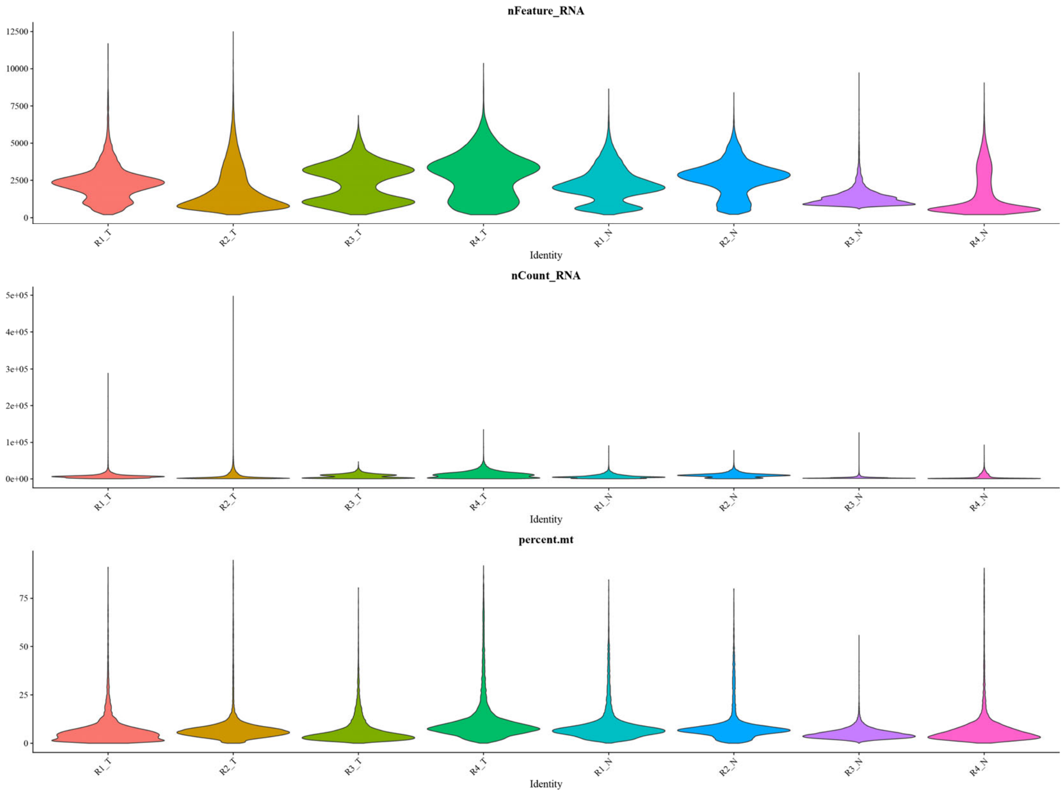

(a)

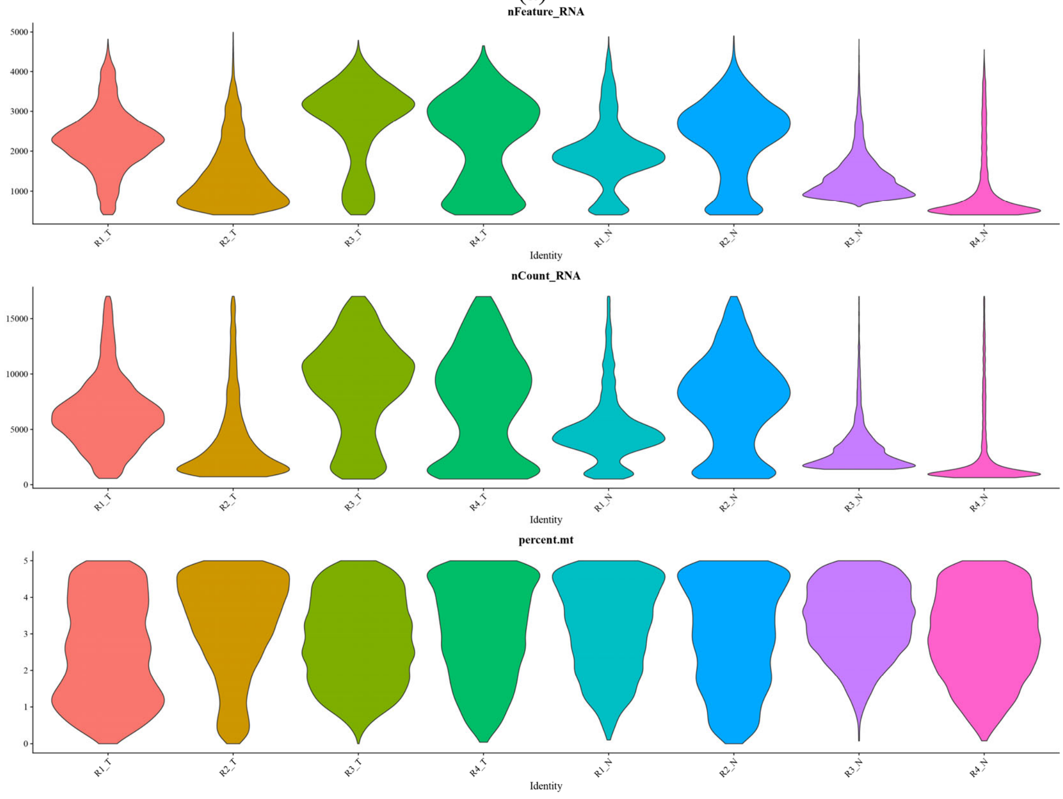

(b)

Figure S1. Quality control of scRNA-seq data. (a) Quality control chart before QC. (b) Quality control chart after QC.

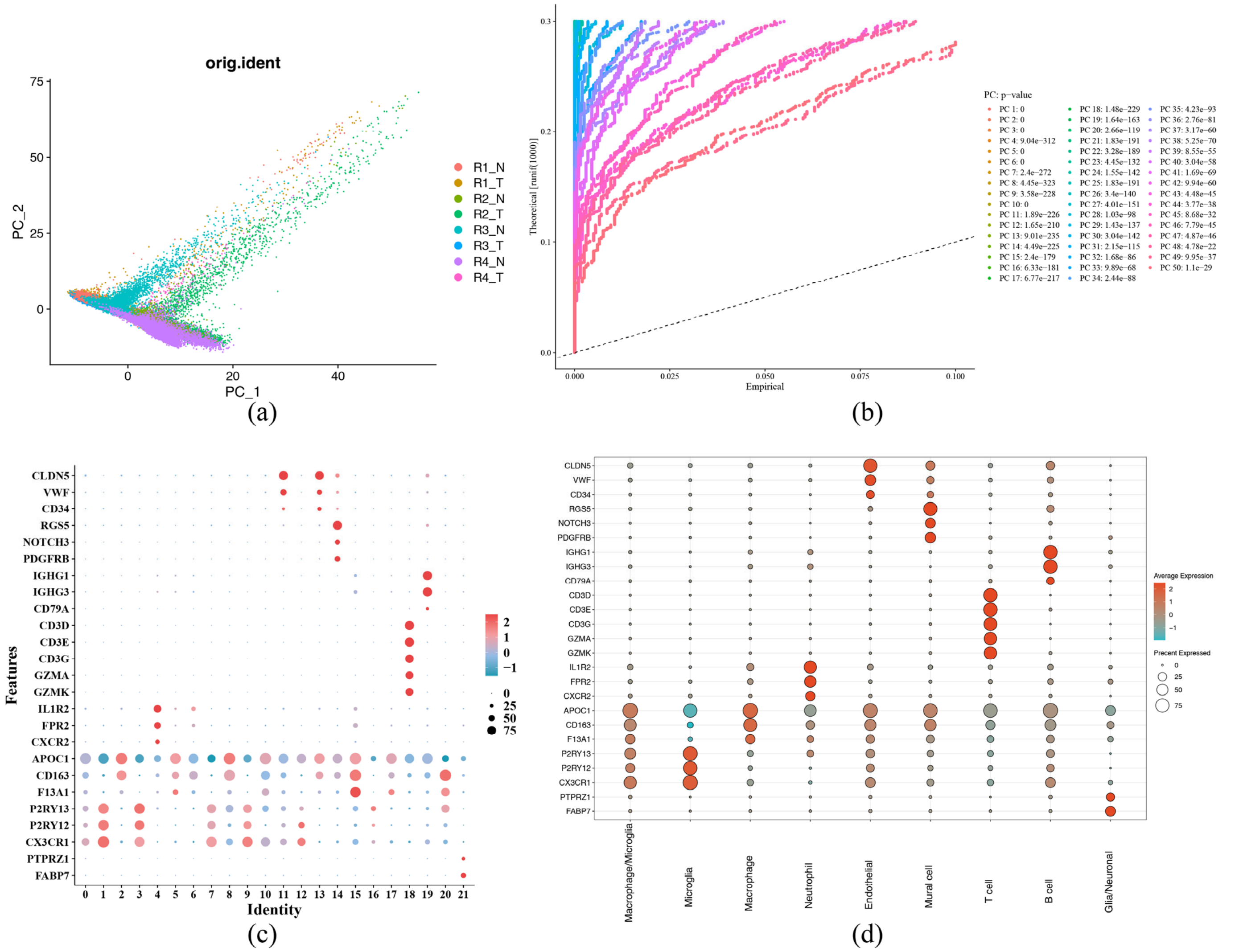

Figure S2. Diagram of principal component analysis and marker gene expression. (a) Scatter plot of PCA. (b) Line chart of PCA. (c) Bubble plot of marker gene expression. (d) Bubble plots of marker gene expression for various cell types.

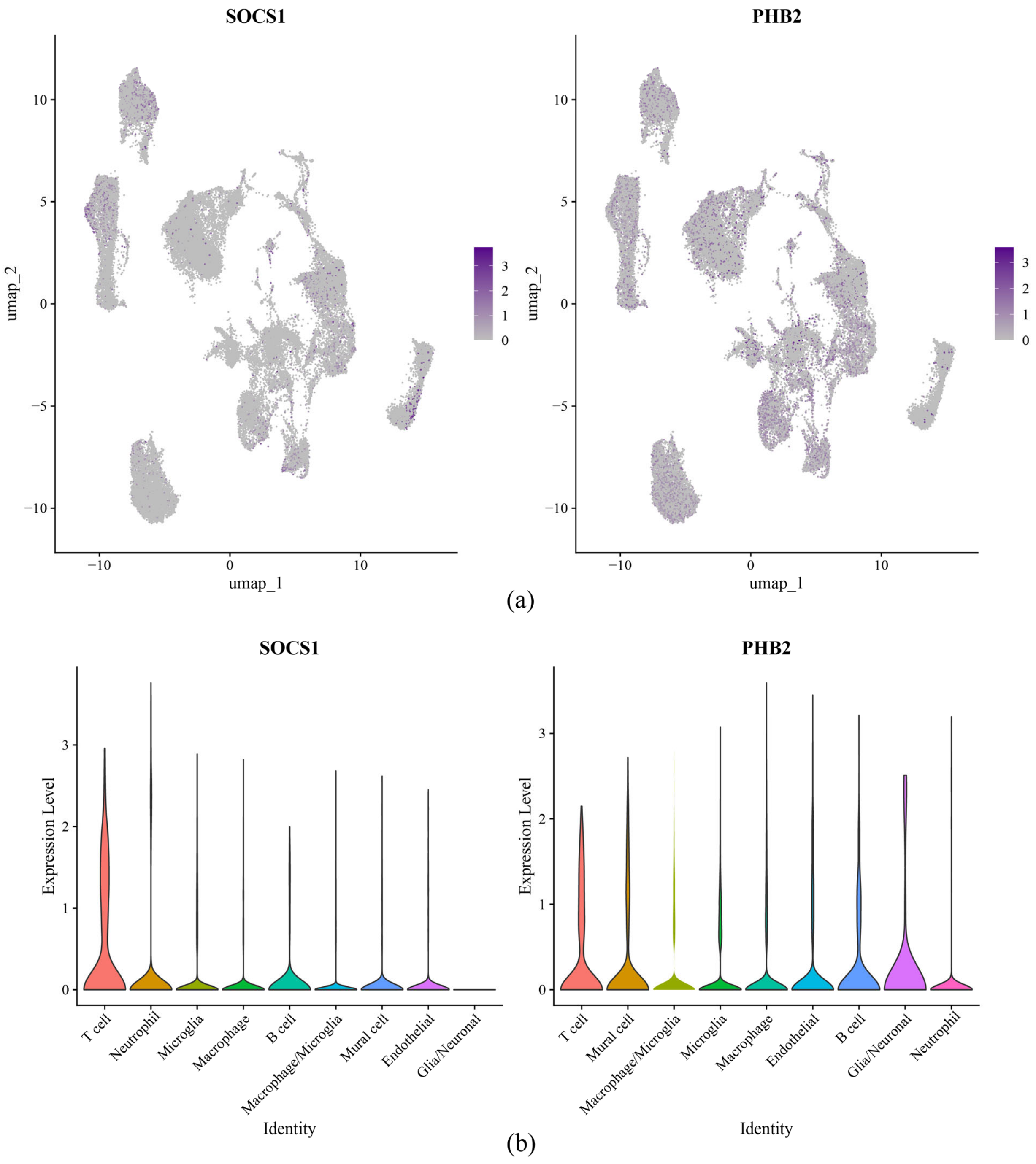

Figure S3. The expression of prognostic genes. (a) The expression of prognostic genes in various cells. (b) The expression of B prognostic genes in different cell clusters.

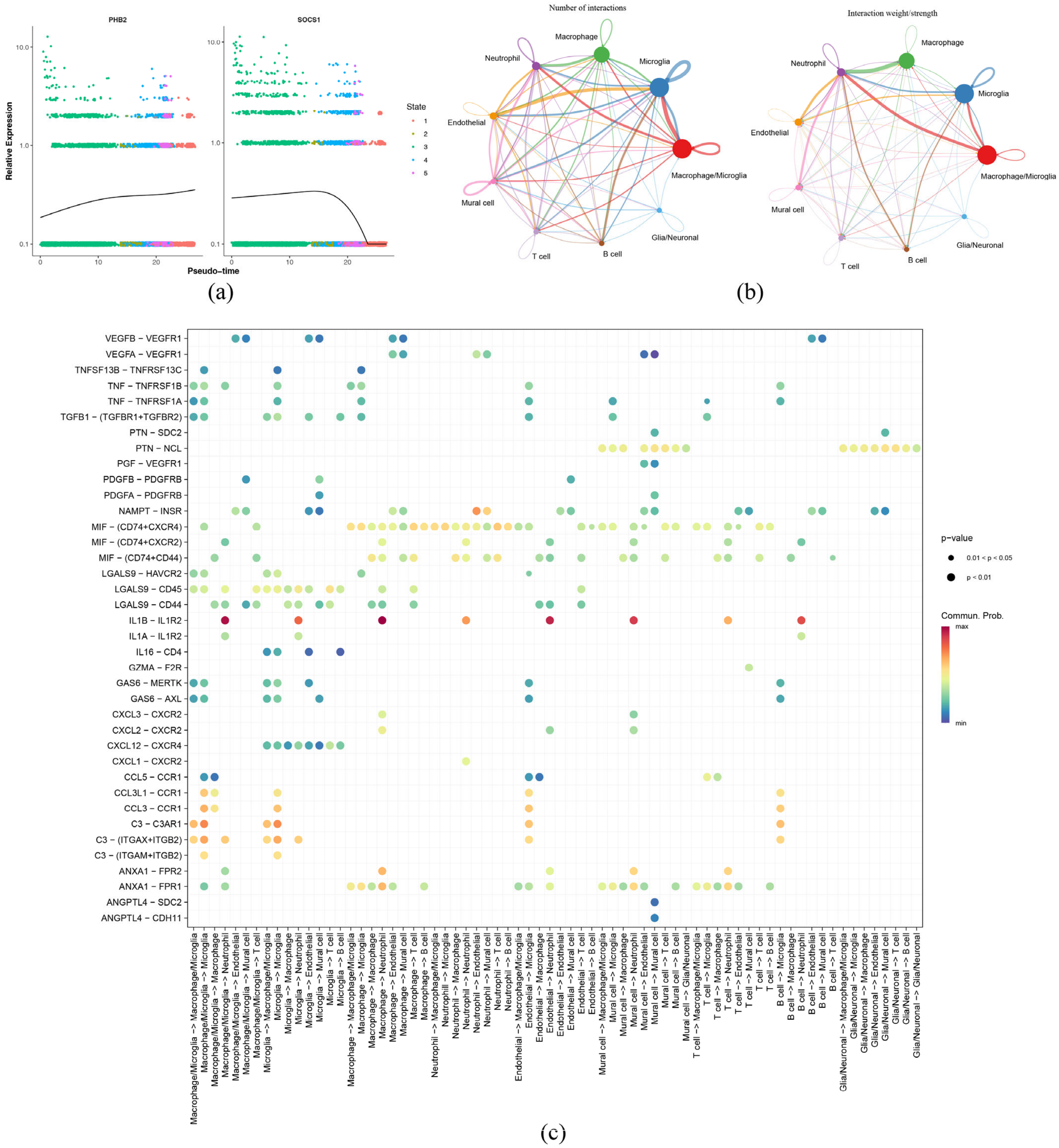

(c)

Figure S4. The results of cell communication and pseudo temporal analysis. (**A**) Expression of prognostic genes in pseudo time. The figure illustrates the expression of prognostic genes over pseudo time, with each panel corresponding to a prognostic gene, emphasizing the dynamic trend of gene expression. (**B**) Network of number of interactions and associated strength of interactions between key cells and other cell types, the left figure shows the network of number of interactions between key cells and other cell types, and the right figure shows the network of interaction strength. The nodes and colors in the figure indicate different cell types, the size of the node circle indicates how many cells of that cell type are involved, and the thickness of the line indicates the number or strength of communication. (**C**) Cell communication signal analysis results. The nodes and colors in

the graph indicate different cell types, the size of the node circle indicates how many cells of that cell type are present, the thickness of the line indicates the number or strength of the communication, and the color of the line corresponds to the color of the cell.
